# Identification and validation of gestational diabetes subgroups by data-driven cluster analysis

**DOI:** 10.1007/s00125-024-06184-7

**Published:** 2024-05-27

**Authors:** Benedetta Salvatori, Silke Wegener, Grammata Kotzaeridi, Annika Herding, Florian Eppel, Iris Dressler-Steinbach, Wolfgang Henrich, Agnese Piersanti, Micaela Morettini, Andrea Tura, Christian S. Göbl

**Affiliations:** 1 CNR Institute of Neuroscience, Padua, Italy; 2 Department of Obstetrics, Charité -Universitätsmedizin Berlin, Corporate Member of Freie Universität Berlin, Humboldt-Universität zu Berlin and Berlin Institute of Health, Berlin, Germany; 3 Department of Obstetrics and Gynaecology, Medical University of Vienna, Vienna, Austria; 4 Department of Information Engineering, Università Politecnica delle Marche, Ancona, Italy; 5 Department of Obstetrics and Gynaecology, Division of Obstetrics, Medical University of Graz, Graz, Austria

**Keywords:** Cluster analysis, Data-driven clustering, Gestational diabetes mellitus, Oral glucose tolerance test, Pregnancy outcomes, Treatment stratification, Unsupervised machine learning

## Abstract

**Aims/hypothesis:**

Gestational diabetes mellitus (GDM) is a heterogeneous condition. Given such variability among patients, the ability to recognise distinct GDM subgroups using routine clinical variables may guide more personalised treatments. Our main aim was to identify distinct GDM subtypes through cluster analysis using routine clinical variables, and analyse treatment needs and pregnancy outcomes across these subgroups.

**Methods:**

In this cohort study, we analysed datasets from a total of 2682 women with GDM treated at two central European hospitals (1865 participants from Charité University Hospital in Berlin and 817 participants from the Medical University of Vienna), collected between 2015 and 2022. We evaluated various clustering models, including *k*-means, *k*-medoids and agglomerative hierarchical clustering. Internal validation techniques were used to guide best model selection, while external validation on independent test sets was used to assess model generalisability. Clinical outcomes such as specific treatment needs and maternal and fetal complications were analysed across the identified clusters.

**Results:**

Our optimal model identified three clusters from routinely available variables, i.e. maternal age, pre-pregnancy BMI (BMIPG) and glucose levels at fasting and 60 and 120 min after the diagnostic OGTT (OGTT0, OGTT60 and OGTT120, respectively). Cluster 1 was characterised by the highest OGTT values and obesity prevalence. Cluster 2 displayed intermediate BMIPG and elevated OGTT0, while cluster 3 consisted mainly of participants with normal BMIPG and high values for OGTT60 and OGTT120. Treatment modalities and clinical outcomes varied among clusters. In particular, cluster 1 participants showed a much higher need for glucose-lowering medications (39.6% of participants, compared with 12.9% and 10.0% in clusters 2 and 3, respectively, *p*<0.0001). Cluster 1 participants were also at higher risk of delivering large-for-gestational-age infants. Differences in the type of insulin-based treatment between cluster 2 and cluster 3 were observed in the external validation cohort.

**Conclusions/interpretation:**

Our findings confirm the heterogeneity of GDM. The identification of subgroups (clusters) has the potential to help clinicians define more tailored treatment approaches for improved maternal and neonatal outcomes.

**Graphical Abstract:**

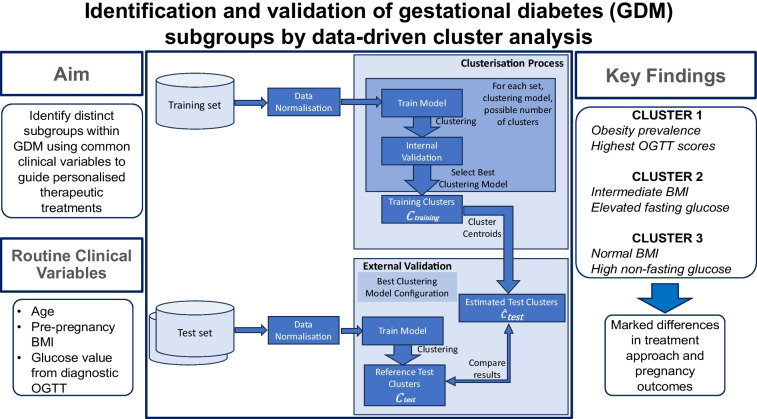

**Supplementary Information:**

The online version of this article (10.1007/s00125-024-06184-7) contains peer-reviewed but unedited supplementary material.


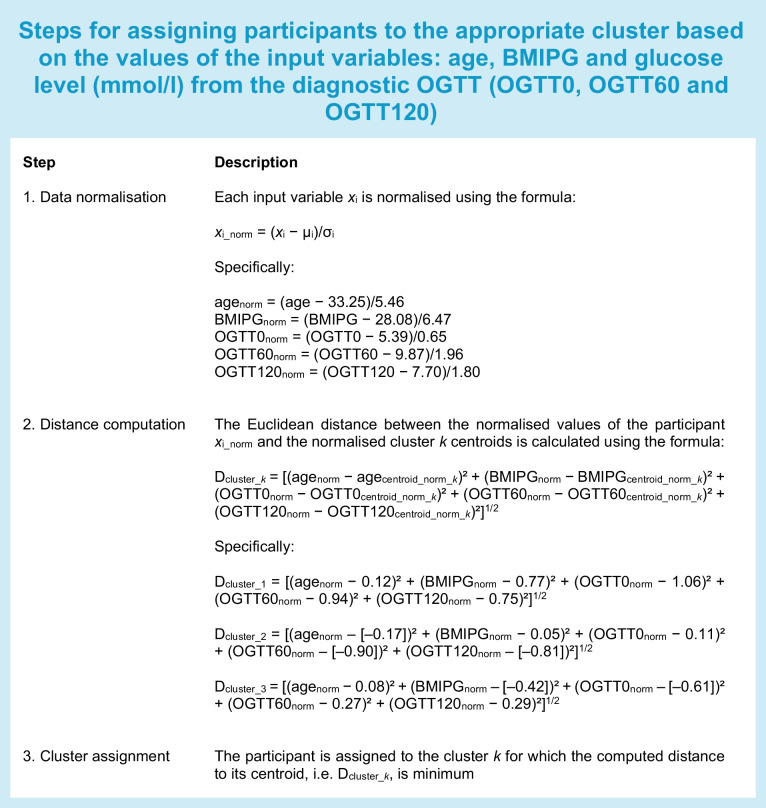



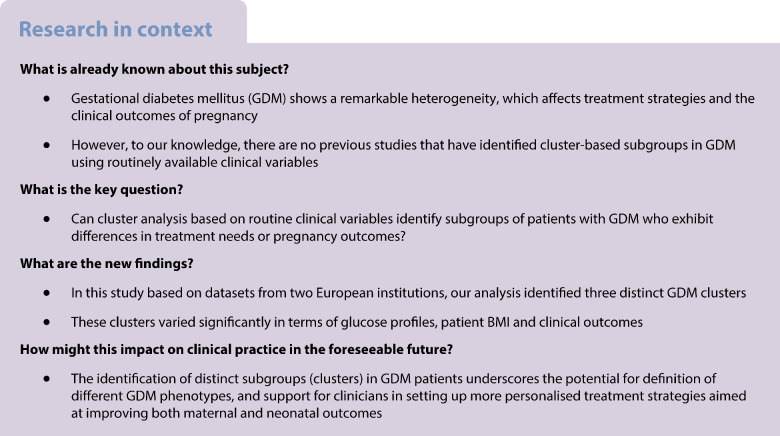


## Introduction

Diabetes mellitus is a heterogeneous disease. Thus, there is currently growing interest in exploiting analytical techniques, particularly unsupervised machine learning, to identify subtypes of patients within those with diabetes that could be the basis for defining more targeted therapeutic strategies [1, 2]. A well-known example of such approach is the study by Ahlqvist et al, which introduced a classification of adult onset diabetes based on cluster analysis [3]. Studies with a similar approach have been performed subsequently [4, 5].

Gestational diabetes mellitus (GDM) is similarly characterised by considerable ‘phenotypic heterogeneity’ [6], which was recently identified as an important research gap by the National Institute of Diabetes and Digestive and Kidney Diseases Workshop [7]. As such, women with GDM may differ in terms of pregnancy outcomes and required treatment strategy. Some women achieve good glycaemic control through diet and lifestyle modification, whereas other women require long-acting insulin (to improve fasting glucose) or short-acting insulin (to achieve acceptable postprandial glucose), and some women will need both short- and long-acting insulin, possibly with further treatment in addition (e.g. metformin). However, to our knowledge, no previous studies have identified GDM subgroups through cluster analysis using only variables commonly measured in the clinical routine of GDM treatment. Therefore, this study aimed to identify clusters of GDM using basic clinical variables and investigate whether the identified clusters are associated with specific treatment needs/modalities or clinical outcomes, including the occurrence of pregnancy complications.

## Methods

### Participants and experimental procedures

**Fig. 1:**
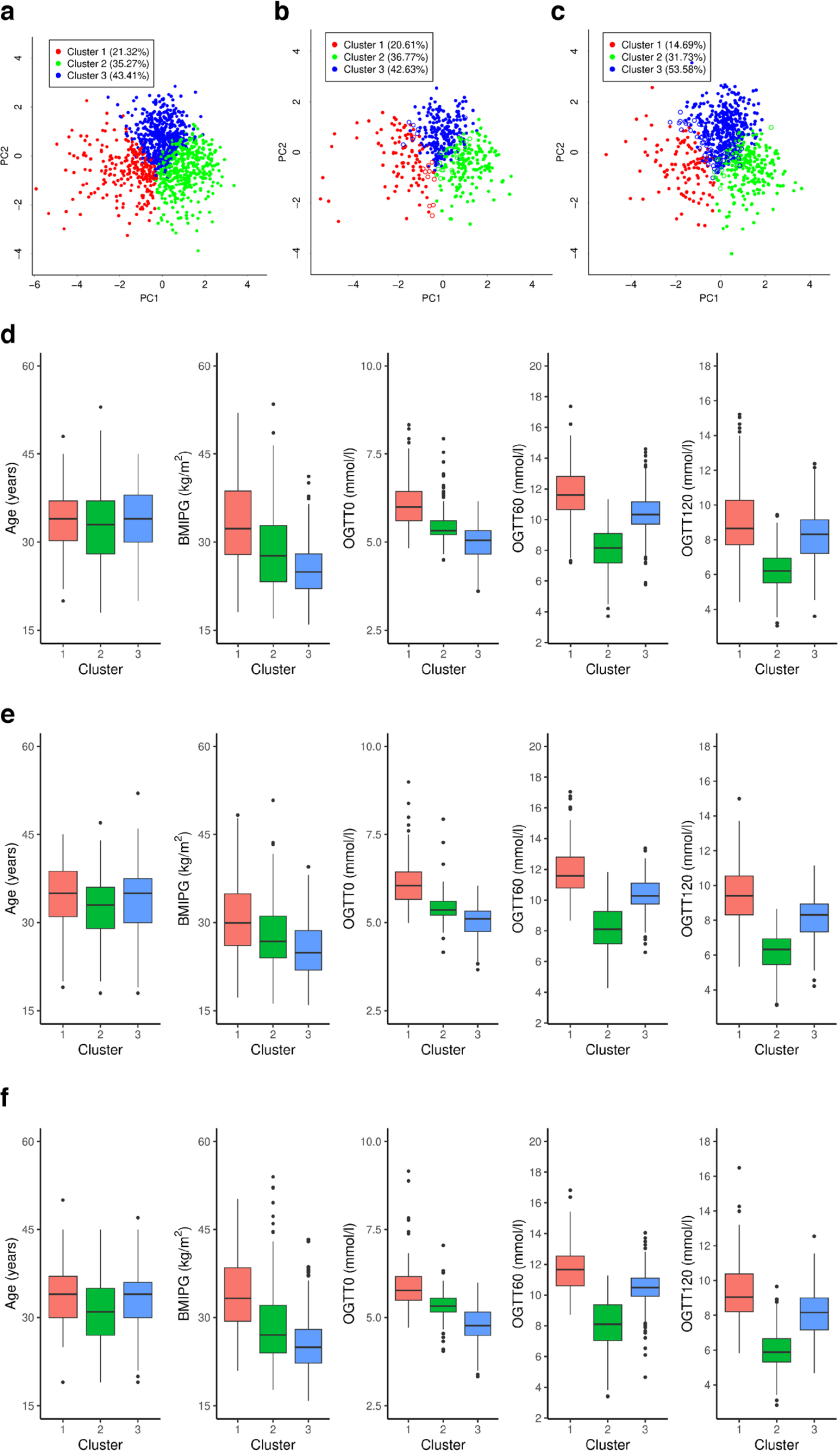
(**a**–**c**) Representation of identified clusters on the principal components space (first two principal components, PC1 and PC2) for the training set (**a**), and of estimated clusters for the Berlin test set (**b**) and Vienna test set (**c**). Each point corresponds to a participant, and different colours represent the assigned cluster; for test sets, filled circles indicate participants correctly assigned to a cluster, whereas empty circles indicate participants assigned to a different cluster than that in the related reference clusters. The percentage of participants assigned to each cluster is also reported. (**d**–**f**) Distribution of input variables in the identified clusters for the training set (**d**), Berlin test set (**e**) and Vienna test set (**f**)

A prospectively compiled dataset was analysed that comprised all singleton pregnancies with GDM diagnosis attending the Pregnancy Outpatient Department at Charité University Hospital (Berlin, Germany) between 2015 and 2022. Another cohort was used for external validation, and comprised women attending the Pregnancy Outpatient Department at the Medical University of Vienna (Vienna, Austria) in the same years. In both cohorts, women with pre-gestational diabetes or with multiple pregnancies were excluded. If any woman had multiple deliveries during the study period, we focused on the first pregnancy. Hence, the final sample consisted of 1865 and 817 women for Berlin and Vienna, respectively. A flow chart with detailed information for included and excluded patients is provided in the electronic supplemental material (ESM) Fig. 1. A list of all variables in Berlin and Vienna datasets is provided in ESM Table 1. Due to the nature of the present study, detailed information about ethnicity and socioeconomic status of the included patients was not available. GDM diagnosis was established by performing a 75 g OGTT in the late second or early third trimester (i.e. between 24 and 28 weeks of gestation), with fasting, 60 and 120 min glucose concentrations equal to or exceeding 5.1, 10 and 8.5 mmol/l (or 92, 180 and 153 mg/dl), respectively [8, 9]. In participants at high risk of GDM or those with elevated fasting glucose concentrations at early pregnancy, the presence of GDM was verified by early OGTT testing before 24 weeks, according to local guidelines [10]. All participants with GDM received lifestyle advice and medical nutrition therapy, and guidance on capillary blood glucose measurement. Glucose‐lowering medication was initiated if the fasting or 1 h postprandial capillary blood glucose exceeded 5.3 or 7.8 mmol/l (95 or 140 mg/dl), with intermediate (or long-acting) insulin being prescribed for elevated fasting glucose and short-acting insulin being prescribed for postprandial hyperglycaemia [11]. The therapeutic approaches and treatment goals in Berlin and Vienna were comparable, as the same guidelines and recommendations are valid in these countries [10]. Metformin was used in some participants, especially insulin-resistant women, in addition to insulin and/or lifestyle modification. A more detailed description about indications for and use of glucose-lowering medication is provided in ESM Methods. The study was approved by the local ethics committees (Berlin: EA2/097/23, Vienna: 1542/2019), and performed in accordance with the Declaration of Helsinki. Further details are reported in ESM Methods.

### Identification and validation of clusters

#### Definition of training and test datasets, input and outcome variables

The pre-processing workflow for deriving training and test sets is described in ESM Methods. The Berlin dataset was randomly partitioned into a training set (70%) and a test set (30%). The Vienna dataset was used as an independent test set for validation of identified clustering models [3]. Different sets of input variables were considered to identify potential clusters. The variable sets were selected with the aim of identifying meaningful clusters while ensuring applicability in routine GDM clinical practice. One set comprised age, pre-pregnancy BMI (BMIPG), and the glucose values from the OGTT (fasting, 60 and 120 min: OGTT0, OGTT60 and OGTT120, respectively). Another set included the same variables excluding age. Further sets included age, BMIPG and either mean OGTT glucose or simply fasting glucose. Outcome variables were defined in relation to clinical outcomes of interest (see ESM Table 1). Table 1 reports summary statistics for the training set, Berlin test set and Vienna test set.

**Table 1:** Summary statistics for the training set, Berlin test set and Vienna test set

	*N*_tot_	Training set	Berlin test set	Vienna test set
Cluster input variables				
Age (years)	2418 (1154/495/769)	33 (30–37)	34 (30–37)	33 (29–36)^*,†^
BMIPG (kg/m^2^)	2418 (1154/495/769)	27.05 (23.10–31.98)	26.70 (23.35–30.80)	26.67 (23.31–31.18)
OGTT0 (mmol/l)	2418 (1154/495/769)	5.33 (5.11–5.72)	5.33 (5.11–5.66)	5.16 (4.72–5.49)^*,†^
OGTT60 (mmol/l)	2418 (1154/495/769)	10.05 (8.60–11.14)	10.05 (8.57–11.10)	10.21 (8.88–10.99)
OGTT120 (mmol/l)	2418 (1154/495/769)	7.60 (6.44–8.77)	7.71 (6.44–8.82)	7.55 (6.27–8.88)
Pharmacological treatment (glucose-lowering medications)				
Drugs prescribed (binary)	2402 (1148/492/762)	209 (18.21)	88 (17.89)	333 (43.70)^*,†^
Pregnancy disorders				
Pre-eclampsia/eclampsia (binary)	2415 (1153/493/769)	66 (5.72)	23 (4.67)	18 (2.34)^*,†^
Maternal delivery complications				
Maternal delivery complications (binary)	2415 (1153/495/767)	606 (52.56)	244 (49.29)	450 (58.67)^*,†^
Pre-term delivery (binary)	2416 (1154/495/767)	60 (5.20)	21 (4.24)	63 (8.21)^*,†^
Delivery <28 weeks (binary)	2416 (1154/495/767)	2 (0.17)	0 (0.00)	2 (0.26)
Assisted delivery (binary)	2415 (1153/495/767)	585 (50.74)	240 (48.48)	431 (56.19)^*,†^
Caesarean section (binary)	2415 (1153/495/767)	495 (42.93)	199 (40.20)	412 (53.72)^*,†^
Primary Caesarean section (binary)	2415 (1153/495/767)	251 (21.77)	103 (20.81)	364 (47.46)^*,†^
Secondary Caesarean section (binary)	2415 (1153/495/767)	244 (21.16)	96 (19.39)	45 (5.87)^*,†^
Vacuum extraction (binary)	2415 (1153/495/767)	90 (7.81)	41 (8.28)	19 (2.48)^*,†^
High base excess (binary)^a^	2033 (936/393/704)	8 (0.85)	3 (0.76)	29 (4.12)^*,†^
Low base excess (binary)^b^	2033 (936/393/704)	725 (77.46)	293 (74.55)	361 (51.28)^*,†^
In-range base excess (binary)	2033 (936/393/704)	203 (21.69)	97 (24.68)	314 (44.60)^*,†^
Fetal complications				
Fetal complications (binary)	2260 (1069/460/731)	607 (56.78)	267 (58.04)	322 (44.05)^*,†^
Birthweight > 4000 g (binary)^c^	2272 (1094/474/704)	155 (14.17)	70 (14.77)	57 (8.10)^*,†^
Birthweight > 4500 g (binary)^c^	2272 (1094/474/704)	28 (2.56)	7 (1.48)	5 (0.71)^*^
LGA (binary)	2412 (1153/495/764)	276 (23.94)	135 (27.27)	115 (15.05)^*,†^
SGA (binary)	2412 (1153/495/764)	86 (7.46)	43 (8.69)	68 (8.90)
Fetal acidosis (binary)^d^	2397 (1150/492/755)	279 (24.26)	101 (20.53)	123 (16.29)^*^
Admission to NICU (binary)	2200 (1016/434/750)	76 (7.48)	45 (10.37)	69 (9.20)
Anomalous APGAR 0 min (binary)^e^	2404 (1147/493/764)	62 (5.41)	21 (4.26)	30 (3.93)
Anomalous APGAR 5 min (binary)^e^	2405 (1148/493/764)	18 (1.57)	5 (1.01)	10 (1.31)
Anomalous APGAR 10 min (binary)^e^	2405 (1148/493/764)	12 (1.05)	2 (0.41)	6 (0.79)
Anomalous APGAR (binary)^f^	2404 (1147/493/764)	66 (5.75)	22 (4.46)	30 (3.93)
Birth percentile				
Birthweight percentile	2412 (1153/495/764)	68.10 (34.82–89.17)	68.68 (36.61–91.36)	54.61 (29.38–80.53)^*,†^
Birth head circumference percentile	2404 (1152/494/758)	81.37 (49.06–95.78)	80.42 (46.99–94.1)	78.62 (47.76–92.44)
Birth length percentile	2411 (1153/495/763)	92.98 (66.64–99.11)	92.98 (66.64–99.46)	84.60 (58.71–98.00)^*,†^
Pharmacological treatment (glucose-lowering medications)^g^				
Metformin only (binary)	762 (0/0/762)	–	–	58 (7.61)
Insulin only (binary)	762 (0/0/762)	–	–	234 (30.71)
Insulin + metformin (binary)	762 (0/0/762)	–	–	41 (5.38)
Metformin (only, or plus insulin) (binary)	763 (0/0/763)	–	–	100 (13.11)
Dose of metformin (mg)	110 (0/0/110)	–	–	1500 (1000–2375)
Insulin (only, or plus metformin) (binary)	766 (0/0/766)	–	–	276 (36.03)
NPH insulin (binary)	765 (0/0/765)	–	–	241 (31.50)
Long-acting insulin (binary)	760 (0/0/760)	–	–	16 (2.11)
Dose of NPH or long-acting insulin (mg)	260 (0/0/260)	–	–	12 (8–22)
Rapid-acting insulin (binary)	763 (0/0/763)	–	–	115 (15.07)
Dose of rapid-acting insulin (mg)	126 (0/0/126)	–	–	10 (4–20)
Dose of total insulin (mg)	107 (0/0/107)	–	–	24 (16–44)
Other patient information				
Number of pregnancies	2412 (1154/495/763)	2 (2–4)	3 (2–4)	3 (2–4)
Number of deliveries	2412 (1154/495/763)	2 (1–3)	2 (1–3)	1 (0–2)^*,†^
GDM history (binary)^g^	762 (0/0/762)	–	–	114 (14.96)
Anti-hypertensive medication (binary)^g^	768 (0/0/768)	–	–	60 (7.81)
Number of anti-hypertensive medications^g^	763 (0/0/763)	–	–	0 (0–0)
Diabetes family history (binary)^g^	763 (0/0/763)	–	–	292 (38.27)

*N*_tot_ is the total number of participants (and their distribution across the training set, Berlin test set and Vienna test set, respectively) for which the corresponding information was available. Categorical data are expressed as *n* (%). Continuous data are presented as median and IQR

^a^Base excess >2

^b^Base excess <−2

^c^For gestational age at birth >37 weeks

^d^Arterial blood pH <7.2

^e^Anomalous if APGAR <7

^f^Anomalous APGAR at 0, 5 or 10 min

^g^Available only for the Vienna test set

Significant differences across sets are indicated as follows: ^*^p< 0.05 between training set and Vienna test set; ^†^*p*< 0.05 between the Berlin test set and the Vienna test set

APGAR, appearance, pulse, grimace, activity, respiration; LGA, large for gestational age; NICU, neonatal intensive care unit; NPH, neutral protamine Hagedorn; SGA, small for gestational age

#### Clustering models implementation and validation

Various clustering algorithms were tested: *k*-means using Euclidean distances, and *k*-medoids and agglomerative hierarchical clustering [12] using both Euclidean and Manhattan distances. A hierarchical clustering algorithm was implemented with three agglomeration methodologies: complete, average and ward.D2 linkages [12]. We also explored the use of two density-based clustering methods: DBSCAN (density-based spatial clustering of applications with noise) [12] and HDBSCAN (hierarchical density-based spatial clustering) [13].

To determine the possible number of clusters for each algorithm and set of input variables, several methods were employed: the Gap statistic method [12], silhouette maximisation [12] and the function NbClust from the ‘NbClust’ R package [14], whereby various indices are calculated and the number of clusters proposed by the majority of indices is selected. Lastly, the elbow method [12] was applied for further heuristic assessment of the potential number of clusters through visualisation of cluster compactness.

We then applied a range of internal validation techniques on the training set to select valid clustering solutions and identify the optimal one. First, we ensured the solution stability (indicated by a Jaccard index above 0.75) and its compactness (indicated by a non-negative mean silhouette) for each cluster [15, 16]. The significance of each input variable was evaluated as discussed below [16]. Moreover, a twofold cross-validation was implemented on two random subsets from the training set [16]. These ‘partial models’ were then compared with the ‘original model’ (i.e. derived from the entire training set) across three key aspects: input similarity, result similarity and outcome significance consistency. With regard to result similarity, several metrics were employed, including the adjusted Rand index and classification metrics such as sensitivity, specificity and the F1 score (see ESM Methods for details) [17].

Following identification and selection of the optimal model, its clustering results underwent an external validation phase on the two test sets to assess generalisability. For a given clustering algorithm and input variables, we defined the training cluster *C*_*training*_ (clustering results from the training set) and the estimated test cluster $\widehat{C}_{\mathrm{test}}$ (clustering results for the test set using the model determined from the training set). In $\widehat{C}_{\mathrm{test}}$, each participant was assigned to the cluster whose centroid was nearest. We also defined the reference test cluster *C*_test_, representing the clustering results on the test set obtained by applying the same clustering algorithm directly to the test set itself. Comparison between *C*_*training*_ and $\widehat{C}_{\mathrm{test}}$ allowed assessment of possible differences in each cluster between the training set and the test sets. Finally, comparison between $\widehat{C}_{\mathrm{test}}$ and *C*_test_ allowed validation of the clustering results on the test sets, evaluating the consistency of cluster assignments [18] (see ESM Methods and ESM Table 2). All procedures were performed in R, version 4.2.2 (https://cran.rstudio.com/). ESM Fig. 2 shows the implemented clustering analysis procedure.

### Statistical analysis

Each input variable was evaluated in terms of significant differences across clusters, this being one of the criteria for acceptance of a potential clustering solution [16]. The normality of variable distribution was checked via a Shapiro–Wilk test and a graphical test of normal distribution. ANOVA followed by Fisher’s protected least significant difference post hoc test was used for normally distributed variables to achieve 95% coverage probability, while the Kruskal–Wallis and Dunn post hoc tests [19] were used for non-normally distributed variables [19]. The *p* values in Tables 2, 3 and 4 were interpreted in an explorative manner.

**Table 2:** Summary statistics for the training set

	*N*_tot_	Cluster 1	Cluster 2	Cluster 3
Cluster input variables				
Age (years)	1154 (246/407/501)	34 (30.25–37)	33 (28–37)^*^	34 (30–38)^‡^
BMIPG (kg/m^2^)	1154 (246/407/501)	32.30 (27.90–38.70)	27.70 (23.25–32.85)^*^	24.90 (22.00–28.00)^†,‡^
OGTT0 (mmol/l)	1154 (246/407/501)	5.99 (5.61–6.44)	5.33 (5.22–5.61)^*^	5.05 (4.66–5.33)^†,‡^
OGTT60 (mmol/l)	1154 (246/407/501)	11.60 (10.66–12.81)	8.16 (7.19–9.10)^*^	10.32 (9.71–11.16)^†,‡^
OGTT120 (mmol/l)	1154 (246/407/501)	8.66 (7.71–10.27)	6.22 (5.52–6.94)^*^	8.32 (7.21–9.16)^†,‡^
Pharmacological treatment (glucose-lowering medications)				
Drugs prescribed (binary)	1016 (212/364/440)	84 (39.62)	47 (12.91)^*^	44 (10.00)^†^
Pregnancy disorders				
Pre-eclampsia/eclampsia (binary)	1153 (246/407/500)	18 (7.32)	27 (6.63)	21 (4.20)
Maternal delivery complications				
Maternal delivery complications (binary)	1153 (246/406/501)	133 (54.07)	205 (50.49)	268 (53.49)
Pre-term delivery (binary)	1154 (246/407/501)	13 (5.28)	18 (4.42)	29 (5.79)
Delivery <28 weeks (binary)	1154 (246/407/501)	0 (0.00)	0 (0.00)	2 (0.40)
Assisted delivery (binary)	1153 (246/406/501)	130 (52.85)	199 (49.01)	256 (51.10)
Caesarean section (binary)	1153 (246/406/501)	118 (47.97)	166 (40.89)	211 (42.12)
Primary Caesarean section (binary)	1153 (246/406/501)	60 (24.39)	93 (22.91)	98 (19.56)
Secondary Caesarean section (binary)	1153 (246/406/501)	58 (23.58)	73 (17.98)	113 (22.55)
Vacuum extraction (binary)	1153 (246/406/501)	12 (4.88)	33 (8.13)	45 (8.98)
High base excess (binary)^a^	936 (216/314/406)	2 (0.93)	3 (0.96)	3 (0.74)
Low base excess (binary)^b^	936 (216/314/406)	155 (71.76)	243 (77.39)	327 (80.54)^†^
In-range base excess (binary)	936 (216/314/406)	59 (27.31)	68 (21.66)	76 (18.72)^†^
Fetal complications				
Fetal complications (binary)	1068 (227/376/465)	140 (61.67)	204 (54.26)	260 (55.91)
Birthweight >4000 g (binary)^c^	1094 (233/389/472)	46 (19.74)	47 (12.08)^*^	62 (13.14)^†^
Birthweight >4500 g (binary)^c^	1094 (233/389/472)	8 (3.43)	11 (2.83)	9 (1.91)
LGA (binary)	1153 (246/406/501)	75 (30.49)	91 (22.41)^*^	110 (21.96)^†^
SGA (binary)	1153 (246/406/501)	15 (6.10)	32 (7.88)	39 (7.78)
Fetal acidosis (binary)^d^	1150 (244/406/500)	63 (25.82)	99 (24.38)	117 (23.40)
Admission to NICU (binary)	1016 (212/364/440)	17 (8.02)	18 (4.95)	41 (9.32)
Anomalous APGAR 0 min (binary)^e^	1147 (245/405/497)	18 (7.35)	20 (4.94)	24 (4.83)
Anomalous APGAR 5 min (binary)^e^	1148 (245/405/498)	6 (2.45)	3 (0.74)	9 (1.81)
Anomalous APGAR 10 min (binary)^e^	1148 (245/405/498)	5 (2.04)	2 (0.49)	5 (1.00)
Anomalous APGAR (binary)^f^	1147 (245/405/497)	19 (7.76)	22 (5.43)	25 (5.03)
Birth percentile				
Birthweight percentile	1153 (246/406/501)	75.83 (47.26–92.62)	67.18 (32.31–88.05)^*^	66.56 (31.87–88.31)^†^
Birth head circumference percentile	1152 (246/405/501)	82.31 (47.76–97.12)	82.31 (47.32–95.76)	80.04 (49.44–95.76)
Birth length percentile	1153 (246/406/501)	94.91 (74.78–99.52)	89.65 (66.64–98.98)^*^	92.78 (66.64–98.98)^†^
Other patient information				
Number of pregnancies	1154 (246/407/501)	3 (2–5)	2 (2–4)^*^	2 (1–4)^†^
Number of deliveries	1154 (246/407/501)	2 (1–4)	2 (1–3)^*^	2 (1–3)^†,‡^

*N*_tot_ is the total number of participants (and their distribution across the training set, Berlin test set and Vienna test set, respectively) for which the corresponding information was available. Categorical data are expressed as *n* (%). Continuous data are presented as median and IQR

^a^Base excess >2

^b^Base excess <−2

^c^For gestational age at birth >37 weeks

^d^Arterial blood pH <7.2

^e^Anomalous if APGAR <7

^f^Anomalous APGAR at 0, 5 or 10 min

Significant differences across sets are indicated as follows: ^*^p<0.05 between cluster 1 and cluster 2; ^†^*p*<0.05 between cluster 1 and cluster 3; ^‡^*p*<0.05 between cluster 2 and cluster 3

APGAR, appearance, pulse, grimace, activity, respiration; LGA, large for gestational age; NICU, neonatal intensive care unit; SGA, small for gestational age

**Table 3:** Summary statistics for the Berlin test set

	*N*_tot_	Cluster 1	Cluster 2	Cluster 3
Cluster input variables				
Age (years)	495 (102/182/211)	35 (31–38.75)	33 (29–36)^*^	35 (30–37.50)^‡^
BMIPG (kg/m^2^)	495 (102/182/211)	29.95 (26.13–34.9)	26.80 (24.02–31.12)^*^	24.90 (21.90–28.65)^†,‡^
OGTT0 (mmol/l)	495 (102/182/211)	6.05 (5.66–6.44)	5.36 (5.22–5.61)^*^	5.11 (4.75–5.33)^†,‡^
OGTT60 (mmol/l)	495 (102/182/211)	11.57 (10.78–12.79)	8.10 (7.16–9.25)^*^	10.27 (9.74–11.10)^†,‡^
OGTT120 (mmol/l)	495 (102/182/211)	9.41 (8.32–10.54)	6.33 (5.45–6.94)^*^	8.32 (7.33–8.94)^†,‡^
Pharmacological treatment (glucose-lowering medications)				
Drugs prescribed – insulin (binary)	435 (90/160/185)	32 (35.56)	20 (12.50)^*^	18 (9.73)^†^
Pregnancy disorders				
Pre-eclampsia/eclampsia (binary)	493 (102/182/209)	3 (2.94)	11 (6.04)	9 (4.31)
Maternal delivery complications				
Maternal delivery complications (binary)	495 (102/182/211)	53 (51.96)	91 (50.00)	100 (47.39)
Pre-term delivery (binary)	495 (102/182/211)	8 (7.84)	6 (3.30)	7 (3.32)
Delivery <28 weeks (binary)	495 (102/182/211)	0 (0.00)	0 (0.00)	0 (0.00)
Assisted delivery (binary)	495 (102/182/211)	52 (50.98)	90 (49.45)	98 (46.45)
Caesarean section (binary)	495 (102/182/211)	45 (44.12)	74 (40.66)	80 (37.91)
Primary Caesarean section (binary)	495 (102/182/211)	26 (25.49)	43 (23.63)	34 (16.11)
Secondary Caesarean section (binary)	495 (102/182/211)	19 (18.63)	31 (17.03)	46 (21.80)
Vacuum extraction (binary)	495 (102/182/211)	7 (6.86)	16 (8.79)	18 (8.53)
High base excess (binary)^a^	393 (84/141/168)	1 (1.19)	1 (0.71)	1 (0.60)
Low base excess (binary)^b^	393 (84/141/168)	63 (75.00)	104 (73.76)	126 (75.00)
In-range base excess (binary)	393 (84/141/168)	20 (23.81)	36 (25.53)	41 (24.40)
Fetal complications				
Fetal complications (binary)	460 (94/167/199)	50 (53.19)	101 (60.48)	116 (58.29)
Birthweight >4000 g (binary)^c^	474 (94/176/204)	13 (13.83)	30 (17.05)	27 (13.24)
Birthweight >4500 g (binary)^c^	474 (94/176/204)	0 (0.00)	2 (1.14)	5 (2.45)
LGA (binary)	495 (102/182/211)	27 (26.47)	55 (30.22)	53 (25.12)
SGA (binary)	495 (102/182/211)	6 (5.88)	10 (5.49)	27 (12.80)^‡^
Fetal acidosis (binary)^d^	492 (100/182/210)	15 (15.00)	37 (20.33)	49 (23.33)
Admission to NICU (binary)	434 (90/160/184)	13 (14.44)	18 (11.25)	14 (7.61)
Anomalous APGAR 0 min (binary)^e^	493 (102/180/211)	6 (5.88)	10 (5.56)	5 (2.37)
Anomalous APGAR 5 min (binary)^e^	493 (102/180/211)	3 (2.94)	0 (0.00)	2 (0.95)
Anomalous APGAR 10 min (binary)^e^	493 (102/180/211)	1 (0.98)	0 (0.00)	1 (0.47)
Anomalous APGAR (binary)^f^	493 (102/180/211)	6 (5.88)	10 (5.56)	6 (2.84)
Birth percentile				
Birthweight percentile	495 (102/182/211)	66.09 (41.22–91.78)	75.39 (44.76–92.31)	65.18 (28.90–89.98)
Birth head circumference percentile	494 (101/182/211)	79.77 (53.36–93.82)	82.31 (53.36–96.16)	76.06 (39.16–93.96)
Birth length percentile	495 (102/182/211)	92.98 (62.74–99.51)	94.24 (79.52–99.49)	91.48 (58.71–98.98)
Other patient information				
Number of pregnancies	495 (102/182/211)	3 (2–5)	2 (2–3)^*^	2 (1–4)^†^
Number of deliveries	495 (102/182/211)	2 (1–3)	2 (1–3)	2 (1–3)

*N*_tot_ is the total number of participants (and their distribution across the training set, Berlin test set and Vienna test set, respectively) for which the corresponding information was available. Categorical data are expressed as *n* (%). Continuous data are presented as median and IQR

^a^Base excess >2

^b^Base excess <−2

^c^For gestational age at birth >37 weeks

^d^Arterial blood pH <7.2

^e^Anomalous if APGAR <7

^f^Anomalous APGAR at 0, 5 or 10 min

Significant differences across sets are indicated as follows: ^*^p<0.05 between cluster 1 and cluster 2; ^†^*p*<0.05 between cluster 1 and cluster 3; ^‡^*p*<0.05 between cluster 2 and cluster 3

APGAR, appearance, pulse, grimace, activity, respiration; LGA, large for gestational age; NICU, neonatal intensive care unit; SGA, small for gestational age

**Table 4:** Summary statistics for the Vienna test set

	*N*_tot_	Cluster 1	Cluster 2	Cluster 3
Cluster input variables				
Age (years)	769 (113/244/412)	34 (30–37)	31 (27–35)^*^	34 (30–36)^‡^
BMIPG (kg/m^2^)	769 (113/244/412)	33.30 (29.36–38.51)	27.04 (24.00–32.11)^*^	24.98 (22.25–27.97)^†,‡^
OGTT0 (mmol/l)	769 (113/244/412)	5.77 (5.49–6.16)	5.33 (5.16–5.55)^*^	4.77 (4.50–5.16)^†,‡^
OGTT60 (mmol/l)	769 (113/244/412)	11.65 (10.60–12.54)	8.10 (7.03–9.38)^*^	10.49 (9.93–11.10)^†,‡^
OGTT120 (mmol/l)	769 (113/244/412)	9.05 (8.21–10.38)	5.88 (5.31–6.66)^*^	8.16 (7.16–9.00)^†,‡^
Pharmacological treatment (glucose-lowering medications)				
Drugs prescribed (insulin/metformin) (binary)	762 (113/242/407)	87 (76.99)	98 (40.50)^*^	148 (36.36)^†^
Metformin only (binary)	762 (113/242/407)	10 (8.85)	18 (7.44)	30 (7.37)
Insulin only (binary)	762 (113/242/407)	54 (47.79)	73 (30.17)^*^	107 (26.29)^†^
Insulin + metformin (binary)	762 (113/242/407)	23 (20.35)	7 (2.89)^*^	11 (2.70)^†^
Metformin (only, or plus insulin) (binary)	763 (113/243/407)	33 (29.20)	26 (10.70)^*^	41 (10.07)^†^
Dose of metformin (mg)	110 (33/27/50)	2000 (1500–2500)	1500 (1000–2000)^*^	1500 (1000–2000)^†^
Insulin (only, or plus metformin) (binary)	766 (113/243/410)	77 (68.14)	80 (32.92)^*^	119 (29.02)^†^
NPH insulin (binary)	765 (113/243/409)	62 (54.87)	78 (32.10)^*^	101 (24.69)^†,‡^
Long-acting insulin (binary)	760 (110/241/409)	11 (10.00)	1 (0.41)^*^	4 (0.98)^†^
Dose of NPH or long-acting insulin (mg)	260 (72/79/109)	17 (10–28)	12 (10–22)	10 (8–16)^†,‡^
Rapid-acting insulin (binary)	763 (113/241/409)	39 (34.51)	16 (6.64)^*^	60 (14.67)^†,‡^
Dose of rapid-acting insulin (mg)	126 (40/21/65)	14 (7.75–21)	6 (2–10)^*^	10 (4–20)^†^
Dose of total insulin (mg)	107 (35/21/51)	38 (24–56)	24 (14–30)^*^	20 (14–36)^†^
Pregnancy disorders				
Pre-eclampsia/eclampsia (binary)	769 (113/244/412)	4 (3.54)	2 (0.82)	12 (2.91)
Maternal delivery complications				
Maternal delivery complications (binary)	767 (113/243/411)	76 (67.26)	153 (62.96)	221 (53.77)^†,‡^
Pre-term delivery (binary)	767 (113/243/411)	7 (6.19)	12 (4.94)	44 (10.71)^‡^
Delivery < 28 weeks (binary)	767 (113/243/411)	0 (0.00)	0 (0.00)	2 (0.49)
Assisted delivery (binary)	767 (113/243/411)	74 (65.49)	149 (61.32)	208 (50.61)^†,‡^
Caesarean section (binary)	767 (113/243/411)	72 (63.72)	144 (59.26)	196 (47.69)^†,‡^
Primary Caesarean section (binary)	767 (113/243/411)	67 (59.29)	131 (53.91)	166 (40.39)^†,‡^
Secondary Caesarean section (binary)	767 (113/243/411)	5 (4.42)	13 (5.35)	27 (6.57)
Vacuum extraction (binary)	767 (113/243/411)	2 (1.77)	5 (2.06)	12 (2.92)
High base excess (binary)^a^	704 (103/221/380)	5 (4.85)	10 (4.52)	14 (3.68)
Low base excess (binary)^b^	704 (103/221/380)	40 (38.83)	104 (47.06)	217 (57.11)^†,‡^
In-range base excess (binary)	704 (103/221/380)	58 (56.31)	107 (48.42)	149 (39.21)^†,‡^
Fetal complications				
Fetal complications (binary)	730 (109/231/390)	49 (44.95)	96 (41.56)	172 (44.10)
Birthweight >4000 g (binary)^c^	704 (106/231/367)	10 (9.43)	21 (9.09)	26 (7.08)
Birthweight >4500 g (binary)^c^	704 (106/231/367)	0 (0.00)	2 (0.87)	3 (0.82)
LGA (binary)	764 (113/241/410)	23 (20.35)	45 (18.67)	47 (11.46)^†,‡^
SGA (binary)	764 (113/241/410)	8 (7.08)	19 (7.88)	41 (10.00)
Fetal acidosis (binary)^d^	755 (112/239/404)	19 (16.96)	31 (12.97)	73 (18.07)
Admission to NICU (binary)	750 (108/240/402)	9 (8.33)	17 (7.08)	43 (10.70)
Anomalous APGAR 0 min (binary)^e^	764 (112/243/409)	5 (4.46)	6 (2.47)	19 (4.65)
Anomalous APGAR 5 min (binary)^e^	764 (112/243/409)	1 (0.89)	3 (1.23)	6 (1.47)
Anomalous APGAR 10 min (binary)^e^	764 (112/243/409)	0 (0.00)	0 (0.00)	6 (1.47)
Anomalous APGAR (binary)^f^	764 (112/243/409)	5 (4.46)	6 (2.47)	19 (4.65)
Birth percentile				
Birthweight percentile	764 (113/241/410)	58.85 (36.82–87.91)	56.28 (32.91–84.16)	52.33 (27.18–76.38)^†,‡^
Birth head circumference percentile	758 (110/241/407)	82.78 (61.96–95.78)	80.42 (46.99–92.44)	72.57 (46.99–91.68)^†^
Birth length percentile	763 (113/240/410)	84.60 (51.91–98.15)	88.64 (61.64–98.00)	84.60 (58.71–96.52)
Other patient information				
Number of pregnancies	763 (112/240/411)	3 (2–4.25)	3 (2–4)^*^	3 (2–4)^†^
Number of deliveries	763 (112/240/411)	2 (1–2)	1 (0–2)^*^	1 (0–2)^†^
GDM history (binary)	762 (111/240/411)	27 (24.32)	39 (16.25)	48 (11.68)^†^
Anti-hypertensive medication (binary)	768 (113/243/412)	19 (16.81)	16 (6.58)^*^	25 (6.07)^†^
Number of anti-hypertensive medications	763 (112/240/411)	0 (0–0)	0 (0–0)^*^	0 (0–0)^†^
Diabetes family history (binary)	763 (112/240/411)	55 (49.11)	98 (40.83)	139 (33.82)^†^

*N*_tot_ is the total number of participants (and their distribution across the training set, Berlin test set and Vienna test set, respectively) for which the corresponding information was available. Categorical data are expressed as *n* (%). Continuous data are presented median and IQR

^a^Base excess >2

^b^Base excess <−2

^c^For gestational age at birth >37 weeks

^d^Arterial blood pH <7.2

^e^Anomalous if APGAR <7

^f^Anomalous APGAR at 0, 5 or 10 min

Significant differences across sets are indicated as follows: ^*^p<0.05 between cluster 1 and cluster 2; ^†^*p*<0.05 between cluster 1 and cluster 3; ^‡^*p*<0.05 between cluster 2 and cluster 3

APGAR, appearance, pulse, grimace, activity, respiration; LGA, large for gestational age; NICU, neonatal intensive care unit; NPH, neutral protamine Hagedorn; SGA, small for gestational age

Similar methodology was applied to compare the continuous outcome variables. For binary outcomes, χ^2^ or Fisher’s exact tests and logistic regression analysis were performed [19]. Logistic regression results further underwent ANOVA (likelihood ratio test), and, if significant, subsequent pairwise comparisons. These analyses on the outcome variables were performed separately on the training and test sets, and on partial datasets derived from twofold cross-validation. Statistical analysis was performed using R, version 4.2.2. For all tests, the two-sided significance level was set to 0.05. All *p* values were interpreted in an explorative manner, with the aim of generating new hypotheses. Therefore, no further adjustment for multiplicity was performed in this study, unless otherwise indicated in the text.

## Results

### Selection of the optimal clustering model configuration

The selected clustering model employed the *k*-means algorithm and identified *k*=3 clusters from age, BMIPG, OGTT0, OGTT60 and OGTT120 as input variables. This configuration was selected based on the performance of the tested models against our implemented internal validation criteria. Specifically, both *k*-medoids and all hierarchical clustering models failed to reach the required threshold of 0.75 for the Jaccard index for each cluster, one of the internal validation criteria that was implemented to deem a proposed solution as acceptable. The reduced stability of these solutions was also confirmed by the unsatisfactory metrics achieved in twofold cross-validation, such as the adjusted Rand index. Use of DBSCAN and HDBSCAN was not pursued further as they yielded unsatisfactory results (see ESM Results for details).

Of note, when using *k*-means, the Gap statistic method suggested *k*=1 for the clustering model, while the silhouette method and the majority of the NbClust indices proposed *k*=2. However, upon further analysis, the *k*=2 clustering solution was not found to meet our internal validation criteria, which require that all variables within a proposed clustering solution must be significantly different across the identified clusters (*p*<0.05). Specifically, age did not exhibit significant differences across the two clusters, leading us to discard this partitioning. Thus, *k*=3, the second option identified by NbClust (with modest differentiation compared with the first option), was considered the best choice.

### Main characteristics of identified clusters

The cluster centroids are listed in ESM Table 3. Figure 1 shows the cluster plot, with the proportion of participants assigned to each cluster (Fig. 1a–c) and the variable distribution within each cluster (Fig. 1d–f).

Cluster 1, which included 246 out of 1154 participants (21.3%), exhibited the highest values across all variables, with the majority of participants being obese before pregnancy and having elevated glucose levels throughout the OGTT. Cluster 2 included 407 participants (35.3%) with lower median age and intermediate BMI, but elevated fasting glucose often exceeding the GDM threshold. Cluster 3 included 501 participants (43.4%) whose age was similar to that of cluster 1, with typically normal BMI and elevated post-load glucose levels (OGTT60 and OGTT120).

### Internal validation results

The three clusters identified showed a high Jaccard index (0.88, 0.86 and 0.86 for clusters 1, 2 and 3, respectively) and non-negative silhouette values. These clusters were validated through implementation of twofold cross-validation, showing robustness and reproducibility for cluster characteristics. Differences in input variables were found in almost all pairs of clusters, confirming the importance of the input variables. Further details of the internal validation results are reported in ESM Results, and goodness-of-fit statistics are reported in ESM Table 4.

### External validation results

#### Assigning the participants in the test sets to clusters

Assignment to the clusters was performed for each participant in each of the two test sets as described below (see text box).

#### Comparison between training set and test sets

The distribution of participants among clusters in the Berlin test set (Fig. 1b) closely mirrored that of the training set (application of a χ^2^ test on the contingency table of cluster assignment proportions led to a *p* value of 0.84). Furthermore, no differences were found in the values of variables between these sets, except for BMIPG and OGTT120 in cluster 1. The Vienna test set (the external validation cohort) showed some differences in cluster proportions (Fig. 1c) compared with the training set, but, despite this, showed interesting results in terms of differences among clusters for some outcome variables, as discussed below.

Further information on distribution of variables across clusters is shown in Fig. 1e,f. Other details of external validation results are reported in ESM Results.

#### Comparison between estimated and reference clusters in test sets ($\bf\widehat{C}_{\mathrm{test}}$ and ***C***_test_)

On the test sets, *k*-means clustering with *k*=3 was reiterated as previously illustrated in the text above, providing the *C*_test_ clusters that served as reference for comparison with the estimated clusters ($\widehat{C}_{\mathrm{test}}$). In the Berlin test set, the Jaccard indices for comparison between $\widehat{C}_{\mathrm{test}}$ and *C*_test_ were 0.87, 0.78 and 0.77, for clusters 1, 2 and 3, respectively, while the Vienna test set (the external cohort) showed indices of 0.80, 0.85 and 0.88, respectively. Figure 1b,c show the cluster assignments for both sets. Overall, the adjusted Rand indices were 0.81 and 0.74 for the Berlin and Vienna test sets, with total accuracies of 93.3% and 90.5%, respectively. Specific goodness-of-fit metrics are reported in ESM Table 5.

### Comparison of clinical outcomes across clusters

Clinical outcomes were first analysed on the training set (Table 2). The need for glucose-lowering medications was higher in cluster 1 (39.6%) compared with clusters 2 (12.9%) and 3 (10.0%) (*p*<0.0001). Likewise, birthweight >4000 g and being large for gestational age (LGA) proportions were also higher in cluster 1 (19.7% and 30.5% for birthweight >4000 g and LGA proportion, respectively) compared with cluster 2 (12.1% and 22.4%, respectively) and cluster 3 (13.1% and 22.0%, respectively) (*p*<0.05). Finally, the low and in-range base excess were significantly different between cluster 1 and cluster 3. The significant differences identified in the training set were further explored in the two partial models obtained through twofold cross-validation (see ESM Tables 6 and 7).

The difference in drug prescription rates between cluster 1 vs clusters 2 and 3 was confirmed in the Berlin test set (*p*<0.0001) (Table 3). The increased drug prescription rates in cluster 1 (*p*<0.0001), the higher proportion of babies who were LGA, the higher values for birthweight percentiles, and the difference in low and in-range base excess in cluster 1 vs cluster 3 were confirmed in the Vienna test set (Table 4).

Some outcomes were available for the external validation cohort (Vienna test set) but not for the Berlin sets. Interestingly, all pharmacological treatment outcomes, except use of metformin only, showed differences across clusters (Table 4). Cluster 1 had higher insulin use (68.1%) than in clusters 2 and 3 (32.9% and 29.0%, respectively; *p*<0.0001). Use of rapid-acting insulin was higher in cluster 1 (34.5%) than in clusters 2 and 3 (6.6% and 14.7%, respectively; *p*<0.0001), and lower in cluster 2 vs cluster 3 (*p*<0.0001). Use of neutral protamine Hagedorn (NPH) insulin was again higher in cluster 1 (54.9%) than in clusters 2 and 3 (32.1% and 24.7%, respectively; *p*<0.0001), and higher in cluster 2 vs cluster 3 (*p*<0.05). Use of long-acting insulin was also higher in cluster 1 (10.0%) than in clusters 2 and 3 (0.4% and 1.0%, respectively; *p*<0.002).

The OR for the training set, the partial models from twofold cross-validation, and the two test sets were calculated as described in ESM Results and are reported in ESM Tables 8–12.

### Assigning a new patient to a cluster

Having defined the clustering model, a new patient may be assigned to the appropriate cluster using the same procedure used to assign the patients in the test sets. The steps for patient assignment are summarised in the text box.

## Discussion

This study aimed to assess clusters of GDM through an unsupervised machine learning technique known as data-driven clustering, using easily accessible clinical variables. We identified three clusters, one of which, cluster 1, exhibited a higher risk for the need for glucose-lowering medications, indicating its potential for targeted intervention strategies. This cluster, characterised by a higher BMI and hyperglycaemia at fasting as well as after oral glucose load, may represent a GDM subgroup with severe glucometabolic impairment and a higher need for pharmacological treatment (required by approximately 40% of patients). Moreover, the identified clusters showed differences in treatment modalities, even between clusters 2 and 3. Basal insulin was typically prescribed to participants in cluster 2, while rapid-acting insulin was more often prescribed in cluster 3. Our finding concerning differences in the need for glucose-lowering medications was extremely robust, as comparable differences between the clusters were observed in both test sets and the validation cohort. Furthermore, our analysis revealed additional differences among the three clusters for various outcome variables associated with pregnancy disorders, maternal delivery and fetal complications, although with lower degree of evidence (in some cases, mostly seen in the external validation cohort). In particular, infants of participants in cluster 3 had lower birthweight percentiles and a lower risk for being LGA, suggesting more favourable pregnancy outcomes. These observations underline the possible clinical importance of our study. On the other hand, it should be acknowledged that the differences in neonatal outcomes among clusters, such as the LGA prevalence, were not totally consistent among all participants in the Berlin and Vienna datasets, which was unexpected given that all participants were adequately treated by the same guidelines and recommendations [10]. Consequently, we consider the identified cluster differences in terms of the need for glucose-lowering medications as a more robust and relevant result, suggesting a more severe phenotype of the disease.

One may question the advantages of identifying clusters rather than developing predictors of the clinical outcomes of interest. In fact, developing a predictor is typically easier than identifying clusters because of the lower risk of methodological flaws. However, predictors usually focus on one outcome (such as pharmacological treatment risk), whereas clustering defines subgroups aiming to identify meaningful phenotypes of the disease, which may comprise several physiological/pathophysiological and clinical characteristics. Thus, the cluster definition may be progressively improved by specification of further characteristics. An example of this is seen in the present study, in which specific information about the type of insulin prescription was available in the external validation cohort (Vienna), and we identified differences among clusters for this important clinical aspect. Conversely, this is difficult to assess with a predictor-based approach. With the predictor-based approach, if an investigator focuses on a new outcome (such as predicting the risk for an event or condition that was not addressed before), it is likely that a completely new predictor must be developed, with no relation with the previous predictors. With the cluster-based approach, future studies may instead add characteristics (including risk for events/conditions) that were not addressed originally. Thus, it is possible to rely on the previously identified clusters without the need to perform the necessary methodological steps for new cluster definition (including collection of sufficiently large datasets). As a fact, compared with predictors, cluster-based approaches have the potential to provide a more holistic view of the disease under investigation [20]. It is also worth noting that, when we investigated the performance of logistic regression analysis (a typical predictor-based approach) using the same datasets (the training set and the Berlin and Vienna test sets) and the same input variables (age, BMI and OGTT glucose levels, either separately or together) for prediction of pharmacological treatment requirements, we obtained unsatisfactory results (details not shown). This suggests that, even for prediction of a single clinical outcome of interest, the prediction-based approach may not be adequate or superior to the cluster-based approach.

Our approach has the advantage of being simple and easily applicable in clinical practice, as the defined GDM clusters relied on only five input variables that are consistently available for patients with GDM (age, BMI and three OGTT glucose levels). Based on this approach, every clinician in charge of patients with GDM will be able to easily categorise each patient into a cluster. This has several clinical implications. For patients assigned to cluster 1, the clinician will gain awareness of the elevated risk of need for glucose-lowering medications and especially high insulin requirements, thus providing an indication of possible aggressive titration needs. On the other hand, patients in cluster 1, evaluated by the clinician as requiring intensive lifestyle intervention, may be trained with specific educational programmes to potentially avoid pharmacological treatment. Likewise, for patients in cluster 2 rather than cluster 3, and vice versa, the clinician will be aware that specific treatment modalities may be preferable, as patients in cluster 3 required a treatment approach based on fast-acting insulin formulations, while intermediate-acting insulin formulations were more often required for patients in cluster 2. Future prospective studies may clarify whether specific interventions (e.g. medical nutrition therapy vs pharmacotherapy) are more or less effective in a specific cluster. On the other hand, further studies to generate different cluster definitions may be also pertinent. In our approach, we aimed to use a minimum number of input variables to ensure wide clinical applicability, but future studies may define clusters based on more input variables, with more restricted clinical applicability but probably an improved ability to predict specific clinical outcomes.

Clinicians may wonder whether the assignment of their patients with GDM to the clusters identified in our study can be deemed reliable. Our methodological procedure was careful, with several alternatives tested to obtain the most accurate results. Furthermore, we validated our findings thoroughly, and, most importantly, using two independent datasets, one of which originated in a different clinic (Vienna) to the training set (Berlin). It is worth noting that, on average, the values of the five input variables exploited for clustering were typically different between the Vienna and Berlin datasets. This may be the common situation for clinicians applying our methodology to assign clusters for their patients. Despite this, the validation results were satisfactory. Thus, it is reasonable to expect correct assignment of new patients to our identified clusters, at least for women in a European setting.

Comparing our findings to prior studies is challenging, as, to our knowledge, our study is the first to identify GDM clusters based on routine clinical variables. In a previous study, we found that fasting hyperglycaemia, either isolated or in combination with post-load hyperglycaemia, was associated with a more frequent need for glucose-lowering medications [21]. In another study, we observed different treatment modalities in participants with GDM and higher BMI, demonstrating increased requirement of insulin [22]. We also applied a supervised learning technique to build a predictor of pharmacological requirements on a subset of the Vienna cohort analysed in the present study, in which the relevant prediction variables were glucose levels at fasting and at 60 min after the OGTT, plus age [23]. These previous findings are essentially consistent with those of the present study. Other investigators developed similar approaches [24–28], again with findings that are typically consistent with ours. However, none of those previous studies used unsupervised learning such as cluster analysis.

The necessary number of observations in relation to the number of input variables to ensure accurate results in cluster analysis has previously been reported to be 70 observations per input variable [29]. This value is higher than that required for supervised machine learning [30, 31], suggesting more challenging requirements for unsupervised vs supervised approaches. As the number of participants per input variable was much higher in our study (230 participants per variable in the training set), and we observed consistent results in training and test datasets (both of which respected the necessary observation/input ratio), we believe that our sample size was certainly adequate. One limitation may be that, among all investigated outcomes, only some showed differences among clusters. On the other hand, we considered only five variables for cluster definition: given that such limited data were exploited for GDM cluster definition, it is plausible that the identified clusters may not yield all the clinical information of possible interest. In fact, the extent of the clinical information provided by our cluster definition aligns reasonably with the limited data required.

In summary, our study identifies novel GDM subgroups through unsupervised machine learning using routine clinical variables. The subgroups derived by cluster analysis showed remarkable differences in terms of glucose-lowering medication needs and treatment modalities (e.g. rapid-acting vs intermediate- or long-acting insulin), which is of major clinical relevance. In general, therefore, our methodology holds promises for guiding personalised treatment strategies and enhancing our understanding of GDM heterogeneity.

### Supplementary Information

Below is the link to the electronic supplementary material.

ESM (PDF 1217 KB)

## Data Availability

The datasets analysed in the current study are available from the corresponding author upon reasonable request. The request must include an appropriate protocol and analysis plan, and institutional approvals must be in place before transfer of any information.

## Abbreviations

BMIPG: Pre-pregnancy BMI
DBSCAN: Density-based spatial clustering of applications with noise
GDM: Gestational diabetes mellitus
HDBSCAN: Hierarchical density-based spatial clustering
LGA: Large for gestational age
OGTT0: Glucose level from the diagnostic OGTT at fasting
OGTT60: Glucose level from the diagnostic OGTT at 60 min
OGTT120: Glucose level from the diagnostic OGTT at 120 min
SGA: Small for gestational age
