# Supplementary material for: Identification and validation of gestational diabetes subgroups by data-driven cluster analysis
Source: Diabetologia. 2024 May 27;67(8):1552–66. doi: 10.1007/s00125-024-06184-7 (PMC11343786; doi:10.1007/s00125-024-06184-7)
Supplement: Supplementary file 1 — ESM (PDF 1217 KB) [file 125_2024_6184_MOESM1_ESM.pdf]

Supplementary Material

Identification and validation of gestational diabetes subgroups by data-driven cluster analysis

Summary

ESM Methods 2

Participants and experimental procedures 2

Identification and validation of clusters 2

Definition of training and test datasets, input and outcome variables 2

Clustering models implementation and validation 3

ESM Results 5

Selection of the optimal clustering model configuration 5

Internal validation results 5

External validation results 5

Comparison between training set and test sets 5

Comparison between estimated and reference clusters in test sets ($\hat{C}_{test}$ and C_{test}) 6

Comparison of clinical outcomes across clusters 6

ESM References 7

ESM Tables 8

ESM Table 1: Variables from the Berlin and Vienna datasets 8

ESM Table 2: Confusion matrix obtained by considering C_{test} as the reference, and the $\hat{C}_{test}$ as the prediction . 11

ESM Table 3: Coordinates of centroids and related normalised coordinates. Mean and standard deviation values used for normalisation of the input variables are also reported 12

ESM Table 4: Cluster goodness-of-fit metrics for the two partial models obtained through twofold cross-validation, using the clusters estimated from the original model as the reference 13

ESM Table 5: Sensitivity, specificity, and F1-score for each cluster on the test sets, with clustering obtained from the model determined on the training set 14

ESM Table 6: Summary statistics for the first partial model of the twofold cross-validation 15

ESM Table 7: Summary statistics for the second partial model of the twofold cross-validation 17

ESM Table 8: Odds Ratios (OR) and 95% confidence intervals for binary outcomes among the three clusters in the training set 19

ESM Table 9: Odds Ratios (OR) and 95% confidence intervals for binary outcomes among the three clusters in the Berlin test set 20

ESM Table 10: Odds Ratios (OR) and 95% confidence intervals for binary outcomes among the three clusters in the Vienna test set 21

ESM Table 11: Odds Ratios (OR) and 95% confidence intervals for binary outcomes among the three clusters in the first partial model of the twofold cross-validation 23

ESM Table 12: Odds Ratios (OR) and 95% confidence intervals for binary outcomes among the three clusters in the second partial model of the twofold cross-validation 24

ESM Figures 25

ESM Fig. 1: Diagram of the data pre-processing steps for the Berlin and Vienna datasets 25

ESM Fig. 2: Diagram of the workflow for the development and validation of the GDM clustering model. 26

ESM Methods

Participants and experimental procedures

The ESM Table 1 reports the variables that are present in the Berlin dataset and/or the Vienna dataset.

According to the German and Austrian practice guidelines the diagnostic OGTT is performed at mid gestation (late second or early third trimester) [1]. In some cases, with high risk of GDM or in those with elevated fasting glucose concentrations at early pregnancy, presence of GDM was verified by early OGTT testing before 24 weeks according to local guidelines. Venous blood samples are taken at fasting and at 60 as well as 120 min after ingestion of oral glucose solution (75 g in 300 ml water). Pre-gestational BMI (BMIPG) was calculated by self-reported weight. This approach is regularly used in epidemiological studies and has highly correlated with technician-measured weight [2, 3]. Calculation of sex- and age-adjusted percentiles of infants birth weight was performed by use of international anthropometric standards. Large (LGA) and small-for-gestational-age (SGA) were defined as birth weight above the 90th or below the 10th Intergrowth percentile, respectively [4, 5].

Intermediate-acting Neutral Protamine Hagedorn (NPH) insulin was started in the evening if ≥2 measurements of fasting glucose were equal to or above 5.3 mmol/l (95 mg/dl) in a period of one week, and rapid-acting insulin analogues (Aspart or Lispro) if ≥2 measurements of 1h postprandial glucose (either after breakfast, lunch or dinner) equal or above 7.8 mmol/l (140 mg/dl) in a period of one week. NPH was started with 6 to 10 IU and increased by 4 to 6 IU (or in case of higher doses), and rapid-acting insulin was started with 2 to 4 IU and increased by 2 to 4 IU if thresholds were not achieved within three days. Long-acting insulin analogues such as glargine (U100/U300) were used as an alternative to NPH if necessary. Patients were trained on insulin management and titration according to their glucose levels. Metformin was used in some cases (especially in insulin resistant obese women).

In both study sites (Berlin and Vienna) different variables were extracted for further analyses: maternal characteristics, such as age, height, weight before pregnancy, singleton or multiple pregnancy, parity and number of deliveries, glucose concentrations at fasting, as well at 60 and 120 min during the diagnostic OGTT, use of glucose-lowering medications, pregnancy disorders, fetal and maternal complications. In the Vienna cohort, we collected specific data on treatment strategies, including use and dosage of insulin and/or metformin. Moreover, we collected details about insulin formulations: intermediate-acting insulin, rapid-acting insulin analogues, or long-acting insulin analogues. Specifically, we considered the maximum doses per day. Moreover, neonatal biometry and outcomes (birth weight, length, and head circumference, gestational age at delivery (GAD), transfer to neonatal intensive care unit, cord blood pH, base excess, APGAR scores), and mode of delivery were extracted by referring to the medical history. Lastly, for Vienna cohort we collected information on previous history of GDM, use and number of anti-hypertensive medicines, and family history of diabetes.

Identification and validation of clusters

Definition of training and test datasets, input and outcome variables

The raw datasets underwent a pre-processing phase, whose steps are summarised in ESM Fig. 1. First, we checked that subjects had at least one glucose value of the diagnostic OGTT equal to or above the threshold values for gestational diabetes (GDM) diagnosis [6]. Thus, in the Berlin dataset, 41 subjects were excluded. The subsequent pre-processing phase involved identification and removal of typographical errors and extreme outliers. Typographical errors were identified via the interquartile range method [7]. Following a similar approach as in the study by Ahlqvist *et al.* [8] extreme outliers

were defined as values that deviated by more than five standard deviations from the mean and they were eliminated only on the Berlin dataset (external validation cohort). If an error or outlier was detected in an input variable, the corresponding subject was excluded. Conversely, if an error or outlier was found in an outcome variable, the variable value variable was deleted. This step resulted in the removal of 8 typos and 6 outliers subjects from the Berlin dataset and 3 typos subjects from the Vienna dataset. Additionally, Local Outlier Factor (LOF) method [9] was applied to the input variables of the Berlin dataset. This method, specifically designed to identify and eliminate density-based outliers, led to the exclusion of 11 more subjects. Of note, subjects with multiple pregnancies (such as twin pregnancies) and subjects missing the required input variables were excluded from the analyses. This led to the exclusion of 72 and 78 subjects respectively from the Berlin dataset, and 1 and 44 subjects respectively from the Vienna test set. After this, the Berlin dataset was partitioned into a training set (70%) and a test set (30%) leading to a training set of 1154 subjects and a first test set of 495; concurrently, the Vienna dataset provided a separate test set composed of 769 subjects. The final pre-processing step involved the normalisation of the input variables to minimize the impact of different measurement scales on the clustering results. Importantly, we performed the normalisation of the training and test sets using only the mean and variance computed from the training set to avoid data leakage [10].

Clustering models implementation and validation

Internal validation

In the validation of a proposed clustering solution, considering the selected input variables, clustering algorithm and number of identified clusters, several validation techniques were sequentially applied. The stability of the solution was initially assessed by confirming that the Jaccard index was greater than 0.75 for each cluster [11, 12]. Subsequently, it was verified that the average silhouette of each cluster was non-negative [12]. The significance of each input variable was evaluated [12], as reported in the statistical analysis section (in the main text).

A twofold cross-validation was then implemented on two random subsets from the training set [12]. These "partial models" were then compared to the "original model" derived from the entire training set across three key aspects: input similarity, outcome significance consistency, and result similarity indices. The input similarity was measured on a variable basis between matching clusters. Using variable mean and standard deviation for each cluster in the original model, the z-score was computed for each subject in the partial model; subsequently, a t-test (or its non-parametric counterpart) evaluated whether each z-score transformed variable in each cluster was not significantly different from zero. To assess the consistency in outcome significance, the analyses conducted on the original model were repeated on the two identified partial models. Lastly, on those subjects included in both the partial and original models, the similarity of the results was evaluated using the Adjusted Rand Index (ARI). In fact, ARI is a measure that spans from 0 to 1 (1 indicating identical match between two clustering outcomes), taking into account the potential randomness in subject partitions [13]. In addition, we computed some classification accuracy metrics such as overall accuracy and, for each cluster, sensitivity, specificity, and F1-score.

Through this comprehensive process, it was possible to select valid clustering solutions, and identify the optimal one in terms of input variables, clustering algorithm and number of clusters. This entire validation process was performed exclusively on the training set.

External Validation

Following identification of the optimal model, its clustering results underwent evaluation on the two independent test sets to assess generalisability. As introduced in the main text, for a particular clustering algorithm and input variables we defined the Training Cluster $C_{training}$ (clustering results obtained from the training set) and the Estimated Test Cluster $\hat{C}_{test}$ (clustering results obtained for the

test set using the model determined from the training set). In $\hat{C}_{test}$, each subject was assigned to the cluster whose centroid was nearest. We also defined the Reference Test Cluster C_{test}, which denotes the clustering results on the test set obtained by applying the same clustering algorithm directly to the test set itself. C_{test} served to assess the robustness of $\hat{C}_{test}$, *i.e.*, the clustering obtained from the training data (see ESM Fig. 2).

The first step of the external validation was the comparison between $C_{training}$ and $\hat{C}_{test}$ to evaluate possible differences in terms of each cluster's percentage of subjects compared to the total subjects' number, and in input variables distribution between corresponding clusters. For every cluster identified, comparisons were performed between the distributions of each input variable in the training and test set, using t-test or the Wilcoxon rank-sum test, depending on whether the distributions were gaussian. A two-sided p-value less than 0.05 was considered statistically significant.

Furthermore, the comparison between $\hat{C}_{test}$ and C_{test} represents the second step of the external validation process, to evaluate the stability of cluster membership. This step assesses the consistency of cluster assignments by comparing the groupings derived from applying the clustering technique to the test set (C_{test}) with those obtained by directly transferring the original clustering to the test set data ($\hat{C}_{test}$), integrating methodological and outcome-based validation strategies [14]. To ensure a meaningful comparison of $\hat{C}_{test}$ and C_{test}, we assessed the stability of the Reference Clusters C_{test} by computing the Jaccard index [11].

A confusion matrix (ESM Table 2) was obtained considering the assignments to the clusters of C_{test} as a reference, and key metrics were calculated for each i-th cluster. Sensitivity ($sens_i$) was determined as $\frac{TP_i}{TP_i+FN_i}$, where TP are True Positives and TN are True Negatives; specificity ($spec_i$) was $\frac{TN_i}{TN_i+F\ \ _i}$, and the F1-score ($F1_i$) was $2\frac{sens_i\ .spec_i}{sens_i+sp\ \ _i}$. The total accuracy was computed as the sum of TP and TN for all clusters, divided by the total number of samples. In addition, ARI was calculated to evaluate the similarity between $\hat{C}_{test}$ and C_{test}, as this index provides further insight into effectiveness of the trained clustering model.

ESM Fig. 2 shows a diagram of the procedure implemented for the clustering analysis procedure.

ESM Results

Selection of the optimal clustering model configuration

With regard to DBSCAN, we found unsatisfactory results. By using a k-distance graph, we plotted the distances of each sample to the k-nearest neighbours, where k ranged from 2 to 10, thereafter we also tested k of 20, 30, and 40. However, DBSCAN mostly returned just one large cluster, or many small clusters. When we tested HDBSCAN we observed similar results. Due to these outcomes, we focused on other clustering algorithms.

Internal validation results

With the selected k-means clustering algorithm, each cluster, whose centroids are reported in ESM Table 3, showed a Jaccard index above 0.86 (precisely, 0.88, 0.86, and 0.86 for Clusters 1, 2, and 3, respectively). All clusters had nonnegative silhouette values, as requested by the implemented validation criterion. A comparison of the input variables across the clusters confirmed the significance of all variables, as mirrored by significant differences in almost all pairs of clusters according to post-hoc tests following ANOVA or Kruskall-Wallis tests, the only exception being age between Clusters 1 and 3.

When the twofold cross-validation was applied for the clustering process, we found remarkable similarities across the cluster characteristics. Upon applying z-score transformation to the subjects in the two partial models based on mean and standard deviation derived from the original (complete) model, the t-tests (or Mann-Whitney) tests generally revealed non-significant differences across the majority of the cluster variables. The only exceptions were in partial model 1, with OGTT120 in Cluster 1, OGTT0 in Clusters 2 and 3, and OGTT60 in Cluster 3. In addition to the indicated comparison between each partial model and the original model, we also assessed significance of input variables in each of the partial models, assessing possible differences among variables between cluster pairs in a given partial model, finding a strong agreement with the original model (all variables except in partial model 1 for age between Clusters 2 and 3, and OGTT120 between Cluster 3 and the other two clusters). A summary of the goodness-of-fit statistics for each cluster in both partial models 1 and 2 is provided in ESM Table 4. Finally, ARI values were equal to 0.82 and 0.92, for partial models 1 and 2, respectively, with overall accuracy reaching 93.8% and 97.2%.

External validation results

Comparison between training set and test sets

For the Berlin test set, the proportion of subjects in each cluster closely parallels that of $C_{training}$ (Fig. 1, main text): 102 subjects (20.6%) were assigned to Cluster 1, 182 subjects (36.8%) to Cluster 2, and 211 subjects (42.6%) to Cluster 3. The chi-squared test on the contingency table of cluster assignment proportions between $C_{training}$ and $\hat{C}_{test}$ yielded a non-significant p-value of 0.84. When comparing the variable values in each cluster between $C_{training}$ and $\hat{C}_{test}$, all tests returned non-significant p-values, except for BMIPG and OGTT120 in Cluster 1 only, suggesting that the input variables in $C_{training}$ and $\hat{C}_{test}$ were virtually the same.

With regard to the Vienna test set, we found marginally different proportions of subjects in the cluster, as compared to $C_{training}$: 113 subjects (14.7%) were assigned to Cluster 1, 244 subjects (31.7%) to Cluster 2, and 412 subjects (53.6%) to Cluster 3 (Fig. 1, main text). In fact, the chi-squared test on the contingency table yielded a significant p-value ($p<0.0001$) in this case. When comparing the variable values in each cluster between $C_{training}$ and this test set, few variables revealed non-significant

p-values (age in Clusters 2 and 3, OGTT0 across all clusters, and OGTT120 in Cluster 2). However, clusters overall properties were very similar to those of $C_{training}$.

Comparison between estimated and reference clusters in test sets ($\hat{C}_{test}$ and C_{test})

A summary of goodness-of-fit metrics obtained on the two test sets is reported in ESM Table 5.

Comparison of clinical outcomes across clusters

The significant differences identified in the training set were further explored in the two partial models obtained through a twofold cross-validation (ESM Tables 6 and 7). The need for pharmacological treatment was found different in Cluster 1 *vs.* Clusters 2 and 3 in both partial models ($p < 0.0001$). Large-for-gestational-age (LGA), Birth Weight > 4000 g, and both the weight and length intergrowth percentiles were different between Cluster 1 *vs.* Clusters 2 and 3 in one of the two partial models. On the other hand, the low base excess and in range base excess outcomes, when comparing Cluster 1 *vs.* Cluster 3, did not exhibit significant differences in either of the two partial models.

To evaluate binary outcomes, we computed Odds Ratios (OR) along with their respective 95% confidence intervals. The OR represents the odds that an outcome will occur given a specific cluster membership, compared to the odds of the outcome occurring without membership to the same cluster. To achieve this, we conducted separate logistic regression analyses for each cluster, where each cluster was considered independently as the focal group in relation to non-membership: in the first logistic regression model, we calculated the odds of the outcome within Cluster 1 relative to the odds outside of Cluster 1. This process was repeated for Cluster 2 and Cluster 3 in their respective models. Thus, each logistic regression model treated its corresponding cluster as an independent reference group for comparison. This approach allowed us to comprehensively assess the impact of cluster membership on the binary outcome. The results of these analyses are reported in ESM Tables 8-12 for the training set, test sets, and each of the twofold cross-validation partial models.

ESM References

1. Schäfer-Graf U, Gembruch U, Kainer F, et al (2018) Gestational Diabetes Mellitus (GDM) – Diagnosis, Treatment and Follow-Up. Guideline of the DDG and DGGG (S3 Level, AWMF Registry Number 057/008, February 2018). Geburtshilfe Frauenheilkd 78(12):1219–1231. https://doi.org/10.1055/a-0659-2596

2. Zhang C, Tobias DK, Chavarro JE, et al (2014) Adherence to healthy lifestyle and risk of gestational diabetes mellitus: prospective cohort study. Bmj 349

3. Rimm EB, Stampfer MJ, Colditz GA, Chute CG, Litin LB, Willett WC (1990) Validity of self-reported waist and hip circumferences in men and women. Epidemiology 466–473

4. Villar J, Ismail LC, Victora CG, et al (2014) International standards for newborn weight, length, and head circumference by gestational age and sex: the Newborn Cross-Sectional Study of the INTERGROWTH-21st Project. The Lancet 384(9946):857–868. https://doi.org/10.1016/S0140-6736(14)60932-6

5. Villar J, Giuliani F, Fenton TR, Ohuma EO, Ismail LC, Kennedy SH (2016) INTERGROWTH-21st very preterm size at birth reference charts. The Lancet 387(10021):844–845

6. International Association of Diabetes and Pregnancy Study Groups Consensus Panel, Metzger BE, Gabbe SG, et al (2010) International association of diabetes and pregnancy study groups recommendations on the diagnosis and classification of hyperglycaemia in pregnancy. Diabetes Care 33(3):676–682. https://doi.org/10.2337/dc09-1848

7. Vinutha HP, Poornima B, Sagar BM (2018) Detection of Outliers Using Interquartile Range Technique from Intrusion Dataset. In: Satapathy SC, Tavares JMRS, Bhateja V, Mohanty JR (eds) Information and Decision Sciences. Springer, Singapore, pp 511–518

8. Ahlqvist E, Storm P, Käräjämäki A, et al (2018) Novel subgroups of adult-onset diabetes and their association with outcomes: a data-driven cluster analysis of six variables. Lancet Diabetes Endocrinol 6(5):361–369. https://doi.org/10.1016/S2213-8587(18)30051-2

9. Breunig MM, Kriegel H-P, Ng RT, Sander J (2000) LOF: identifying density-based local outliers. In: Proceedings of the 2000 ACM SIGMOD international conference on Management of data. pp 93–104

10. Hastie T, Tibshirani R, Friedman JH (2009) The elements of statistical learning: data mining, inference, and prediction, 2nd edn. Springer, New York

11. Hennig C (2007) Cluster-wise assessment of cluster stability. Comput Stat Data Anal 52(1):258–271

12. Tkaczynski A (2017) Segmentation using two-step cluster analysis. In: Dietrich T, Rundle-Thiele S, Kubacki K (eds) Segmentation in social marketing. Springer, Singapore, pp 109–125. https://doi.org/10.1007/978-981-10-1835-0_8

13. Hubert L, Arabie P (1985) Comparing partitions. J Classif 2:193–218

14. Ullmann T, Hennig C, Boulesteix A-L (2022) Validation of cluster analysis results on validation data: A systematic framework. Wiley Interdiscip Rev Data Min Knowl Discov 12(3):e1444

ESM Tables

ESM Table 1: Variables from the Berlin and Vienna datasets

VARIABLE LABEL	VARIABLE EXPLANATION	BERLIN DATA	VIENNA DATA
	INPUT VARIABLES CONSIDERED FOR CLUSTERING		
Age (years)	Age at booking (the first appearance of a pregnant women at the outpatient clinic)	X	X
BMIPG (kg/m^2)	BMI pre-gestation	X	X
WeightPG (kg)	Weight pre-gestation	X	X
Height (cm)	Height	X	X
OGTT0 (mmol/l)	Basal Glucose	X	X
OGTT60 (mmol/l)	Glucose at 60 min after glucose load	X	X
OGTT120 (mmol/l)	Glucose at 120 min after glucose load	X	X
Mean OGTT (mmol/l)	Mean glucose value between OGTT0, OGTT60, OGTT120	X	X
	OUTCOMES		
	Drug Treatment Information		
Drugs Prescribed (binary)	Insulin/Metformin/Insulin+Metformin vs. None	X	X
Metformin only (binary)	Metformin vs. None/Metformin+Insulin/Insulin	-	X
Insulin only (binary)	Insulin vs. None/Metformin+Insulin/Metformin	-	X
Insulin + Metformin (binary)	Metformin+Insulin vs. None/Insulin/Metformin	-	X
Metformin (only, or plus insulin) (binary)	Metformin/Metformin+Insulin vs. None/Insulin	-	X
Dose of Metformin (mg)	Prescripted Dose of Metformin	-	X
Insulin (only, or plus metformin) (binary)	Insulin/Metformin+Insulin vs. None/Metformin	-	X
NPH Insulin (binary)	NPH Insulin use	-	X
Long-acting Insulin (binary)	Long-acting (Glargin/Detemir)	-	X
Dose of NPH or Long-acting Insulin (mg)	Prescripted Dose of NPH/Long-acting Insulin	-	X
Rapid-acting Insulin (binary)	Rapid-acting Insulin use	-	X
Dose of Rapid-acting Insulin (mg)	Prescripted Dose of Rapid-acting Insulin	-	X
Dose of Total Insulin (mg)	Prescripted total Insulin dose	-	X
	Pregnancy Disorders		
Preeclampsia / Eclampsia (binary)	0: No; 1: Preeclampsia / Eclampsia	X	X
	***Maternal Delivery Complication*s**		
Maternal Delivery Complications (binary)	Pretermine Delivery or Assisted Delivery	X	X
Pre-term Delivery (binary)	DeliveryGA < 37 weeks	X	X
Delivery < 28 weeks (binary)	DeliveryGA < 28 weeks	X	X
Assisted Delivery (binary)	Nonspontaneous Delivery: Caesarean Section/Vacuum Extraction/Forceps Delivery	X	X
Caesarean Section (binary)	Primary/Secondary Caesarean Section	X	X
Primary Caesarean Section (binary)	Method of assisted delivery	X	X

VARIABLE LABEL	VARIABLE EXPLANATION	BERLIN DATA	VIENNA DATA
Secondary Caesarean Section (binary)	Method of assisted delivery	X	X
Vacuum Extraction / Forceps Delivery (binary)	Method of assisted delivery	X	-
Vacuum Extraction (binary)	Method of assisted delivery	-	X
High Base Excess (binary)	Base Excess > 2	X	X
Low Base Excess (binary)	Base excess < -2	X	X
In Range Base Excess (binary)	-2 ≤ Base Excess ≤ 2	X	X
	Fetal Complications		
Fetal Complications (binary)	Birth Weight > 4000 g / LGA / SGA / Fetal Acidosis / Anomalous APGAR (Appearance, Pulse, Grimace, Activity, Respiration)	X	X
Birth Weight > 4000 g (binary)	If GAdelivery > 37 weeks and birth weight > 4 kg	X	X
Birth Weight > 4500 g (binary)	If GAdelivery > 37 weeks and birth weight > 4.5 kg	X	X
LGA (binary)	Large-For-Gestational-Age (birth weight percentile > 90 intergrowth percentile)	X	X
SGA (binary)	Small-For-Gestational-Age (birth weight percentile < 10 intergrowth percentile)	X	X
Fetal Acidosis (binary)	Arterial blood pH < 7.2	X	X
Admission to NICU admission (binary)	Neonatal Intensive Care Unit Admission	X	X
Anomalous APGAR 0 min (binary)	APGAR 0 min<7	X	X
Anomalous APGAR 5 min (binary)	APGAR 5 min<7	X	X
Anomalous APGAR 10 min (binary)	APGAR 10 min<7	X	X
Anomalous APGAR (binary)	Anomalous APGAR 0 min / Anomalous APGAR 5 min / Anomalous APGAR 10 min	X	X
Fetal Intergrowth Percentiles (%)	Fetal Intergrowth Percentiles (at delivery)	X	X
Birth Weight Percentile	Birth Weight Percentile	X	X
Birth Head Circumference Percentile	Birth Head Circumference Percentile	X	X
Birth Length Percentile	Birth Length Percentile	X	X
	OTHER'S PATIENTS INFORMATION		
Number of Pregnancies (count)	Number of Pregnancies (including present)	X	X
Number of Deliveries (count)	Number of Deliveries (excluding present)	X	X
GDM History (binary)	Previous History of GDM	-	X
Anti-Hypertensive medication (binary)	Use of anti-hypertensive medication	-	X
Number of Anti-Hypertensive medication (count)	Number of used anti-hypertensive medication	-	X
Diabetes Family History (binary)	Family history of diabetes	-	X
	OTHER VARIABLES NECESSARY FOR OUTCOME VARIABLE COMPUTATION		
GAdelivery (weeks)	Gestational age at delivery	X	X
Delivery mode (categorical)	Spontaneous / Primary Section /Secondary Section / (Vacuum Extraction) For Vienna / (Forceps Delivery / Vacuum Extraction) for Berlin	X	X
Rapid-acting Insulin type (categorical)	0: No 1: Humalog 2: Novorapid 3: Tresiba	-	X

VARIABLE LABEL	VARIABLE EXPLANATION	BERLIN DATA	VIENNA DATA
Hypertensive pregnancy disorders (categorical)	No / Pre-existent / Sih (Superimposed Pre-Eclampsia), Occurred In Pregnancy / Preeclampsia / HELLP (Hemolysis, Elevated Liver enzymes and Low Platelets) / Eclampsia	-	X
APGAR 0 min score (0-10 scale)	APGAR 0 min	X	X
APGAR 5 min score (0-10 scale)	APGAR 5 min	X	X
APGAR 10 min score (0-10 scale)	APGAR 10 min	X	X
Arterial cord pH (mmol/l)	Arterial cord pH	X	X
Base Excess (mmol/l)	Base Excess	X	X
Newborn sex (categorical)	Newborn sex	X	X
Fetal weight at delivery (g)	Fetal weight at delivery	X	X
Fetal length at delivery (cm)	Fetal length at delivery	X	X
Fetal head circumference at delivery (cm)	Fetal head circumference at delivery	X	X
	UNUSED VARIABLES		
Gravida (count)	Total number of pregnancies	X	-
Para (count)	Total number of births	X	-
Mutiple pregnancy (binary)	Multiple Pregnancies, e.g., twin pregnancy	X	X
WeightLA (kg)	Last available weight	X	X
BMILA (kg/m^2)	Last available BMI	-	X
WeightLA-GA (weeks)	WeightLA -Gestational Age	-	X
WeightBD (kg)	Weight before delivery	X	-
BMIBD (kg/m^2)	BMI before delivery	X	-
Weight gain (kg)	WeightBD - WeightPG	X	-
preDM (categorical)	0-GDM 1;-DM I; 2-DM II	-	X
GDM.diagnosis.through.path.OGTT (binary)	All =1	-	X
GDM.OGTT.diagnosis (binary)	All =1	-	X
GDM (binary)	All =1	-	X
OGTT gestational age (weeks)	Gestational Age in weeks at OGTT	X	X
OGTT gestational age days (days)	Total Gestational Age at OGTT is equal to OGTT gestational age (weeks) plus OGTT gestational age days (days)	X	-
Nicotin use (binary)	Smoking	-	X
IUFD (binary)	Intrauterine fetal death	X	X
Alterations in cardiotocography (binary)	Alterations in cardiotocography	X	-
Premature rupture of membranes (binary)	Premature rupture of membranes	X	-
Intrauterine growth restriction (binary)	Intrauterine growth restriction	X	-
Emergency section (binary)	Emergency section	X	-
Blood loss during delivery (...)	Blood loss during delivery	X	-
Shoulder dystocia (binary)	Fetus stack from shoulders	X	-
Birth weight int. Percentile	Internal Percentile	-	X
Birth length int. Percentile	Internal Percentile	-	X
Birth head circumference int. Percentile	Internal Percentile	-	X

BMI: body mass index; OGTT: oral glucose tolerance test; LGA: Large-For-Gestational-Age; SGA: Small-For-Gestational-Age; Admission to NICU: Neonatal Intensive Care Unit; NPH: Neutral Protamine Hagedorn; APGAR: Appearance, Pulse, Grimace, Activity, Respiration; DM: diabetes mellitus; GDM: gestational diabetes.

ESM Table 2: Confusion matrix obtained by considering C_{test} as the reference, and the $\hat{C}_{test}$ as the prediction

		Reference Test Cluster (C_{test})	
		Positive (Cluster i)	*Negative (Cluster ≠i)*
Estimated Test Cluster ($\hat{C}_{test}$)	*Positive (Cluster i)*	TP_i	FP_i
	Negative (Cluster ≠i)	FN_i	TN_i

TP: True Positive; FP: Fasle Positive; FN: False Negative; TN: True Negative.

ESM Table 3: Coordinates of centroids and related normalised coordinates. Mean and standard deviation values used for normalisation of the input variables are also reported

	Age	*BMIPG*	*OGTT0*	*OGTT60*	*OGTT120*
Coordinates of centroids and normalised centroids					
Cluster 1	33.91 (years)	33.07 (kg/m^2)	6.07 (mmol/l)	11.71 (mmol/l)	9.06 (mmol/l)
	0.12 (unitless)	0.77 (unitless)	1.06 (unitless)	0.94 (unitless)	0.75 (unitless)
Cluster 2	32.33 (years)	28.43 (kg/m^2)	5.46 (mmol/l)	8.10 (mmol/l)	6.24 (mmol/l)
	-0.17 (unitless)	0.05 (unitless)	0.11 (unitless)	-0.90 (unitless)	-0.81 (unitless)
Cluster 3	33.66 (years)	25.34 (kg/m^2)	4.99 (mmol/l)	10.40 (mmol/l)	8.23 (mmol/l)
	0.08 (unitless)	-0.42 (unitless)	-0.61 (unitless)	0.27 (unitless)	0.29 (unitless)
Mean and standard deviation for normalisation of input variables					
Mean (μ)	33.25 (years)	28.08 (kg/m^2)	5.39 (mmol/l)	9.87 (mmol/l)	7.70 (mmol/l)
Standard deviation (σ)	5.46 (years)	6.47 (kg/m^2)	0.65 (mmol/l)	1.96 (mmol/l)	1.80 (mmol/l)

BMIPG: pre-pregnancy body mass index; OGTT0, OGTT60, OGTT120: glucose from the diagnostic oral glucose tolerance test at fasting: 60, 120 min.

ESM Table 4: Cluster goodness-of-fit metrics for the two partial models obtained through twofold cross-validation, using the clusters estimated from the original model as the reference

	Partial Model 1			*Partial Model 2*		
	Sensitivity	***Specificity***	***F1-score***	***Sensitivity***	***Specificity***	***F1-score***
Cluster 1	88.90 %	98.40 %	91.60 %	93.70 %	100.00 %	96.70 %
Cluster 2	93.50 %	98.10 %	94.90 %	99.50 %	96.50 %	96.70 %
Cluster 3	96.70 %	93.40 %	94.00 %	96.90 %	99.10 %	97.80 %

ESM Table 5: Sensitivity, specificity, and F1-score for each cluster on the test sets, with clustering obtained from the model determined on the training set

	Berlin Test Set (estimated clustering, $\hat{C}_{test}$)			*Vienna Test Set (estimated clustering, $\hat{C}_{test}$)*		
	Sensitivity	***Specificity***	***F1-score***	***Sensitivity***	***Specificity***	***F1-score***
Cluster 1	93.80 %	97.20 %	91.50 %	68.10 %	100.00 %	81.00 %
Cluster 2	96.60 %	95.60 %	94.40 %	92.50 %	98.40 %	94.60 %
Cluster 3	90.60 %	97.00 %	93.30 %	99.70 %	84.60 %	91.30 %

ESM Table 6: Summary statistics for the first partial model of the twofold cross-validation

	N_{tot}	Cluster 1	Cluster 2	Cluster 3
Cluster Input Variables				
Age (years)	577 (127/193/257)	35 [31 - 38]	33 [29 - 37] *	33 [30 - 37] †
BMIPG (kg/m^2)	577 (127/193/257)	33.1 [28.70 - 38.95]	27.30 [23.10 - 32] *	25.70 [22.50 - 29] †,‡
OGTT0 (mmol/l)	577 (127/193/257)	5.94 [5.61 - 6.35]	5.33 [5.16 - 5.55] *	5.11 [4.77 - 5.44] †,‡
OGTT60 (mmol/l)	577 (127/193/257)	11.43 [10.54 - 12.71]	8.16 [6.94 - 9.05] *	10.49 [9.99 - 11.32] †,‡
OGTT120 (mmol/l)	577 (127/193/257)	8.49 [7.44 - 9.99]	6.05 [5.38 - 6.83] *	8.38 [7.21 - 9.21] ‡
Pharmacological Treatment (glucose-lowering medications)				
Drugs prescribed - Insulin (binary)	574 (127/193/254)	50 (39.37)	20 (10.36) *	36 (14.17) †
Pregnancy Disorders				
Preeclampsia / Eclampsia (binary)	577 (127/193/257)	11 (8.66)	7 (3.63)	9 (3.50)
Maternal Delivery Complications				
Maternal Delivery Complications (binary)	577 (127/193/257)	64 (50.39)	92 (47.67)	135 (52.53)
Preterm Delivery (binary)	577 (127/193/257)	8 (6.30)	11 (5.70)	14 (5.45)
Delivery < 28 weeks (binary)	577 (127/193/257)	0 (0.00)	0 (0.00)	1 (0.39)
Assisted Delivery (binary)	577 (127/193/257)	62 (48.82)	86 (44.56)	133 (51.75)
Caesarean Section (binary)	577 (127/193/257)	55 (43.31)	76 (39.38)	106 (41.25)
Primary Caesarean Section (binary)	577 (127/193/257)	31 (24.41)	40 (20.73)	47 (18.29)
Secondary Caesarean Section (binary)	577 (127/193/257)	24 (18.90)	36 (18.65)	59 (22.96)
Vacuum Extraction/Forceps Delivery (binary)	577 (127/193/257)	7 (5.51)	10 (5.18)	27 (10.51)
High Base Excess (binary) [a]	479 (112/149/218)	1 (0.89)	0 (0.00)	1 (0.46)
Low Base Excess (binary) [b]	479 (112/149/218)	81 (72.32)	119 (79.87)	174 (79.82)
In range Base Excess (binary)	479 (112/149/218)	30 (26.79)	30 (20.13)	43 (19.72)
Fetal Complications				
Fetal Complications (binary)	530 (115/181/234)	71 (61.74)	100 (55.25)	129 (55.13)
Birth Weight > 4000 g (binary) [c]	544 (119/182/243)	20 (16.81)	29 (15.93)	33 (13.58)
Birth Weight > 4500 g (binary) [c]	544 (119/182/243)	2 (1.68)	6 (3.30)	2 (0.82)
LGA (binary)	576 (127/192/257)	34 (26.77)	49 (25.52)	58 (22.57)
SGA (binary)	576 (127/192/257)	4 (3.15)	14 (7.29)	19 (7.39)
Fetal Acidosis (binary) [d]	574 (125/192/257)	35 (28.00)	47 (24.48)	56 (21.79)
Admission to NICU (binary)	504 (109/178/217)	12 (11.01)	8 (4.49)	17 (7.83)
Anomalous APGAR 0 min (binary) [e]	574 (126/192/256)	7 (5.56)	11 (5.73)	15 (5.86)
Anomalous APGAR 5 min (binary) [e]	574 (126/192/256)	3 (2.38)	3 (1.56)	6 (2.34)
Anomalous APGAR 10 min (binary) [e]	574 (126/192/256)	3 (2.38)	2 (1.04)	2 (0.78)
Anomalous APGAR (binary) [f]	574 (126/192/256)	7 (5.56)	13 (6.77)	16 (6.25)
Birth Percentile				
Birth Weight at Delivery Percentile	576 (127/192/257)	75.72 [43.67 - 91.03]	69.81 [35.37 - 90.82]	66.69 [31.87 - 88.28]
Birth Head Circumference at Delivery Percentile	576 (127/192/257)	80.42 [46.99 - 95.78]	83.97 [49.95 - 95.78]	79.77 [46.99 - 94.90]

	N_{tot}	Cluster 1	Cluster 2	Cluster 3
Birth Length at Delivery Percentile	576 (127/192/257)	94.24 [74.03 - 99.52]	92.13 [62.1 - 98.15]	92.78 [69.07 - 99.11]
Other Patient Information				
Number of Pregnancies (count)	577 (127/193/257)	3 [2 - 5]	2 [2 - 4] *	2 [2 - 4] †
Number of Deliveries (count)	577 (127/193/257)	3 [1 - 4]	2 [1 - 3] *	2 [1 - 3] †

N_{tot} is the total number of subjects (and their distribution across clusters) for which the corresponding information was available. Categorical data are expressed as n (%). Continuous data are presented as median and IQR.

[a]Base excess >2

[b]Base excess <−2

[c]For gestational age at birth >37 weeks

[d]Arterial blood pH <7.2

[e]Anomalous if APGAR <7

[f]Anomalous APGAR at 0, 5 or 10 min

Significant differences across sets are indicated as follows: $^{*}p<0.05$ between cluster 1 and cluster 2; $^{\dagger}p<0.05$ between cluster 1 and cluster 3; $^{\ddagger}p<0.05$ between cluster 2 and cluster 3

APGAR, appearance, pulse, grimace, activity, respiration; LGA Large for gestational age; NICU, neonatal intensive care unit; SGA, small for gestational age

ESM Table 7: Summary statistics for the second partial model of the twofold cross-validation

	N_{tot}	Cluster 1	Cluster 2	Cluster 3
Cluster Input Variables				
Age (years)	577 (104/220/253)	34 [30 - 38]	32 [28 - 36.25] [*]	34 [31 - 38] [‡]
BMIPG (kg/m^2)	577 (104/220/253)	32.95 [27.78 - 39.40]	27.30 [22.90 - 32.80] [*]	24.9 [22.20 - 28] [†,‡]
OGTT0 (mmol/l)	577 (104/220/253)	6.11 [5.65 - 6.55]	5.38 [5.22 - 5.72] [*]	4.94 [4.61 - 5.27] [†,‡]
OGTT60 (mmol/l)	577 (104/220/253)	11.74 [10.77 - 12.88]	8.21 [7.21 - 9.21] [*]	10.27 [9.71 - 11.04] [†,‡]
OGTT120 (mmol/l)	577 (104/220/253)	8.69 [7.83 - 10.50]	6.38 [5.70 - 7.05] [*]	8.32 [7.33 - 9.10] [†,‡]
Pharmacological Treatment (glucose-lowering medications)				
Drugs prescribed - Insulin (binary)	574 (104/217/253)	36 (34.62)	35 (16.13) [*]	32 (12.65) [†]
Pregnancy Disorders				
Preeclampsia / Eclampsia (binary)	576 (104/220/252)	9 (8.65)	18 (8.18)	12 (4.76)
Maternal Delivery Complications				
Maternal Delivery Complications (binary)	576 (104/219/253)	64 (61.54)	116 (52.97)	135 (53.36)
Preterm Delivery (binary)	577 (104/220/253)	4 (3.85)	8 (3.64)	15 (5.93)
Delivery < 28 weeks (binary)	577 (104/220/253)	0 (0.00)	0 (0.00)	1 (0.40)
Assisted Delivery (binary)	576 (104/219/253)	63 (60.58)	115 (52.51)	126 (49.8)
Caesarean Section (binary)	576 (104/219/253)	59 (56.73)	92 (42.01) [*]	107 (42.29) [†]
Primary Caesarean Section (binary)	576 (104/219/253)	28 (26.92)	55 (25.11)	50 (19.76)
Secondary Caesarean Section (binary)	576 (104/219/253)	31 (29.81)	37 (16.89) [*]	57 (22.53)
Vacuum Extraction/Forceps Delivery (binary)	576 (104/219/253)	4 (3.85)	23 (10.50)	19 (7.51)
High Base Excess (binary) [a]	457 (92/169/196)	1 (1.09)	4 (2.37)	1 (0.51)
Low Base Excess (binary) [b]	457 (92/169/196)	67 (72.83)	127 (75.15)	157 (80.10)
In range Base Excess (binary)	457 (92/169/196)	24 (26.09)	38 (22.49)	38 (19.39)
Fetal Complications				
Fetal Complications (binary)	539 (98/202/239)	63 (64.29)	109 (53.96)	135 (56.49)
Birth Weight > 4000 g (binary) [c]	550 (100/212/238)	24 (24)	19 (8.96) [*]	30 (12.61) [†]
Birth Weight > 4500 g (binary) [c]	550 (100/212/238)	6 (6.00)	5 (2.36)	7 (2.94)
LGA (binary)	577 (104/220/253)	37 (35.58)	44 (20.00) [*]	54 (21.34) [†]
SGA (binary)	577 (104/220/253)	9 (8.65)	16 (7.27)	24 (9.49)
Fetal Acidosis (binary) [d]	576 (104/220/252)	25 (24.04)	55 (25.00)	61 (24.21)
Admission to NICU (binary)	512 (89/192/231)	5 (5.62)	11 (5.73)	23 (9.96)
Anomalous APGAR 0 min (binary) [e]	573 (104/219/250)	9 (8.65)	11 (5.02)	9 (3.60)
Anomalous APGAR 5 min (binary) [e]	574 (104/219/251)	2 (1.92)	0 (0.00)	4 (1.59)
Anomalous APGAR 10 min (binary) [e]	574 (104/219/251)	2 (1.92)	0 (0.00)	3 (1.20)
Anomalous APGAR (binary) [f]	573 (104/219/250)	10 (9.62)	11 (5.02)	9 (3.60)
Birth Percentile				
Birth Weight at Delivery Percentile	577 (104/220/253)	79.68 [47.49 - 96.30]	63.91 [32.66 - 85.36] [*]	66.93 [32.55 - 88.34] [†]
Birth Head Circumference at Delivery Percentile	576 (104/219/253)	83.97 [58.80 - 97.98]	82.31 [53.36 - 93.62]	82.31 [53.36 - 95.78]
Birth Length at Delivery Percentile	577 (104/220/253)	96.81 [74.54 - 99.72]	89.65 [66.88 - 99.02] [*]	92.98 [56.49 - 98.15] [†]

	N_{tot}	Cluster 1	Cluster 2	Cluster 3
Others Patient Information				
Number of Pregnancies (count)	577 (104/220/253)	3 [2 - 4]	2 [1 - 4]	2 [1 - 4]
Number of Deliveries (count)	577 (104/220/253)	2 [1 - 3]	2 [1 - 3]	2 [1 - 3] †

N_{tot} is the total number of subjects (and their distribution across clusters) for which the corresponding information was available. Categorical data are expressed as n (%). Continuous data are presented as median and IQR.

[a]Base excess >2

[b]Base excess <−2

[c]For gestational age at birth >37 weeks

[d]Arterial blood pH <7.2

[e]Anomalous if APGAR <7

[f]Anomalous APGAR at 0, 5 or 10 min

Significant differences across sets are indicated as follows:

*$p<0.05$ between cluster 1 and cluster 2; †$p<0.05$ between cluster 1 and cluster 3; ‡$p<0.05$ between cluster 2 and cluster 3

APGAR, appearance, pulse, grimace, activity, respiration; LGA Large for gestational age; NICU, neonatal intensive care unit; SGA, small for gestational age

ESM Table 8: Odds Ratios (OR) and 95% confidence intervals for binary outcomes among the three clusters in the training set

	Cluster 1	Cluster 2	Cluster 3
Pharmacological Treatment (glucose-lowering medications)			
Drugs Prescribed - Insulin	3.595 [2.601 - 4.968]	0.604 [0.432 - 0.844]	0.542 [0.394 - 0.745]
Pregnancy Disorders			
Preeclampsia / Eclampsia	1.413 [0.806 - 2.476]	1.288 [0.776 - 2.137]	0.592 [0.348 - 1.008]
Maternal Delivery Complications			
Maternal Delivery Complications	1.080 [0.814 - 1.433]	0.88 [0.691 - 1.121]	1.069 [0.846 - 1.349]
Pre-term Delivery	1.022 [0.544 - 1.921]	0.777 [0.441 - 1.368]	1.233 [0.733 - 2.074]
Delivery < 28 weeks	0.000 [0.000 - Inf]	0.000 [0.000 - Inf]	68792797.473 [0.000 - Inf]
Assisted Delivery	1.113 [0.840 - 1.476]	0.899 [0.706 - 1.145]	1.026 [0.813 - 1.295]
Caesarean Section	1.296 [0.977 - 1.720]	0.879 [0.688 - 1.123]	0.943 [0.745 - 1.193]
Primary Caesarean Section	1.209 [0.868 - 1.685]	1.108 [0.828 - 1.481]	0.793 [0.596 - 1.055]
Secondary Caesarean Section	1.196 [0.855 - 1.673]	0.738 [0.544 - 1.002]	1.158 [0.872 - 1.539]
Vacuum Extraction/Forceps Delivery	0.545 [0.292 - 1.018]	1.071 [0.685 - 1.674]	1.331 [0.865 - 2.048]
High Base Excess [a]	1.112 [0.223 - 5.550]	1.190 [0.283 - 5.013]	0.782 [0.186 - 3.290]
Low Base Excess [b]	0.669 [0.473 - 0.946]	0.994 [0.719 - 1.375]	1.373 [1.002 - 1.881]
In range Base Excess	1.503 [1.059 - 2.134]	0.997 [0.717 - 1.386]	0.731 [0.531 - 1.006]
Fetal Complications			
Fetal Complications	1.323 [0.979 - 1.787]	0.868 [0.674 - 1.118]	0.946 [0.741 - 1.207]
Birth Weight > 4000 g [c]	1.697 [1.161 - 2.482]	0.760 [0.526 - 1.097]	0.860 [0.608 - 1.216]
Birth Weight > 4500 g [c]	1.495 [0.650 - 3.439]	1.178 [0.546 - 2.540]	0.617 [0.277 - 1.376]
LGA	1.541 [1.126 - 2.108]	0.878 [0.659 - 1.169]	0.824 [0.625 - 1.085]
SGA	0.765 [0.430 - 1.360]	1.098 [0.697 - 1.731]	1.087 [0.699 - 1.690]
Fetal Acidosis [d]	1.112 [0.803 - 1.539]	1.010 [0.762 - 1.339]	0.920 [0.700 - 1.209]
Admission to NICU	1.101 [0.627 - 1.931]	0.533 [0.309 - 0.919]	1.588 [0.993 - 2.539]
Anomalous APGAR 0 min [e]	1.546 [0.877 - 2.727]	0.866 [0.501 - 1.496]	0.817 [0.483 - 1.381]
Anomalous APGAR 5 min [e]	1.864 [0.692 - 5.018]	0.362 [0.104 - 1.258]	1.311 [0.516 - 3.327]
Anomalous APGAR 10 min [e]	2.667 [0.839 - 8.476]	0.364 [0.079 - 1.668]	0.932 [0.294 - 2.953]
Anomalous APGAR [f]	1.529 [0.880 - 2.658]	0.911 [0.538 - 1.543]	0.787 [0.472 - 1.312]

[a]Base Excess > 2,
[b]Base Excess < -2,
[c]for gestational age at birth > 37 weeks,
[d]Arterial blood pH < 7.2,
[e]Anomalous if APGAR < 7,
[f]Anomalous APGAR at 0 or 5 or 10 min.
APGAR, appearance, pulse, grimace, activity, respiration; LGA Large for gestational age; NICU, neonatal intensive care unit; SGA, small for gestational age

ESM Table 9: Odds Ratios (OR) and 95% confidence intervals for binary outcomes among the three clusters in the Berlin test set

	Cluster 1	Cluster 2	Cluster 3
Pharmacological Treatment (glucose-lowering medications)			
Drugs Prescribed - Insulin	2.551 [1.536 - 4.235]	0.590 [0.354 - 0.982]	0.777 [0.484 - 1.249]
Pregnancy Disorders			
Preeclampsia / Eclampsia	0.562 [0.164 - 1.93]	1.603 [0.692 - 3.711]	0.868 [0.368 - 2.045]
Maternal Delivery Complications			
Maternal Delivery Complications	1.144 [0.74 - 1.769]	1.046 [0.726 - 1.507]	0.876 [0.613 - 1.251]
Pre-term Delivery	2.488 [1.002 - 6.175]	0.677 [0.258 - 1.778]	0.662 [0.262 - 1.669]
Delivery < 28 weeks	1.000 [0.000 - Inf]	1.000 [0.000 - Inf]	1.000 [0.000 - Inf]
Assisted Delivery	1.134 [0.733 - 1.753]	1.063 [0.738 - 1.532]	0.867 [0.607 - 1.239]
Caesarean Section	1.225 [0.789 - 1.903]	1.031 [0.710 - 1.495]	0.847 [0.588 - 1.219]
Primary Caesarean Section	1.404 [0.843 - 2.339]	1.304 [0.838 - 2.031]	0.599 [0.379 - 0.945]
Secondary Caesarean Section	0.939 [0.538 - 1.640]	0.783 [0.488 - 1.257]	1.305 [0.834 - 2.041]
Vacuum Extraction/Forceps Delivery	0.778 [0.334 - 1.810]	1.110 [0.576 - 2.140]	1.058 [0.556 - 2.016]
High Base Excess [a]	1.849 [0.166 - 20.643]	0.893 [0.080 - 9.933]	0.668 [0.060 - 7.425]
Low Base Excess [b]	1.030 [0.591 - 1.797]	0.937 [0.585 - 1.501]	1.042 [0.658 - 1.650]
In range Base Excess	0.942 [0.535 - 1.656]	1.074 [0.667 - 1.728]	0.974 [0.613 - 1.549]
Fetal Complications			
Fetal Complications	0.78 [0.495 - 1.231]	1.171 [0.795 - 1.724]	1.018 [0.701 - 1.480]
Birth Weight > 4000 g [c]	0.909 [0.475 - 1.742]	1.325 [0.792 - 2.218]	0.805 [0.479 - 1.354]
Birth Weight > 4500 g [c]	0.000 [0.000 - Inf]	0.674 [0.129 - 3.509]	3.367 [0.647 - 17.53]
LGA	0.950 [0.581 - 1.555]	1.261 [0.841 - 1.892]	0.826 [0.552 - 1.237]
SGA	0.601 [0.247 - 1.467]	0.493 [0.237 - 1.026]	2.458 [1.288 - 4.690]
Fetal Acidosis [d]	0.628 [0.345 - 1.143]	0.981 [0.623 - 1.544]	1.346 [0.868 - 2.088]
Admission to NICU	1.646 [0.825 - 3.286]	1.160 [0.617 - 2.180]	0.582 [0.300 - 1.128]
Anomalous APGAR 0 min [e]	1.567 [0.592 - 4.145]	1.615 [0.672 - 3.881]	0.404 [0.145 - 1.120]
Anomalous APGAR 5 min [e]	5.894 [0.972 - 35.754]	0.000 [0.000 - Inf]	0.890 [0.147 - 5.374]
Anomalous APGAR 10 min [e]	3.861 [0.239 - 62.258]	0.000 [0.000 - Inf]	1.338 [0.083 - 21.516]
Anomalous APGAR [f]	1.465 [0.558 - 3.844]	1.475 [0.624 - 3.487]	0.487 [0.187 - 1.265]

[a]Base Excess > 2,
[b]Base Excess < -2,
[c]for gestational age at birth > 37 weeks,
[d]Arterial blood pH < 7.2,
[e]Anomalous if APGAR < 7,
[f]Anomalous APGAR at 0 or 5 or 10 min.
APGAR, appearance, pulse, grimace, activity, respiration; LGA Large for gestational age; NICU, neonatal intensive care unit; SGA, small for gestational age

ESM Table 10: Odds Ratios (OR) and 95% confidence intervals for binary outcomes among the three clusters in the Vienna test set

	Cluster 1	Cluster 2	Cluster 3
Pharmacological Treatment (glucose-lowering medications)			
Drugs Prescribed - Insulin / Metformin	5.482 [3.440 - 8.735]	0.825 [0.606 - 1.125]	0.525 [0.393 - 0.702]
Metformin only	1.216 [0.596 - 2.479]	0.964 [0.541 - 1.720]	0.929 [0.544 - 1.588]
Insulin only	2.385 [1.587 - 3.583]	0.963 [0.691 - 1.342]	0.640 [0.470 - 0.873]
Insulin + Metformin	8.959 [4.653 - 17.249]	0.426 [0.186 - 0.975]	0.301 [0.149 - 0.610]
Metformin	3.589 [2.226 - 5.788]	0.722 [0.449 - 1.162]	0.564 [0.368 - 0.864]
Insulin	4.880 [3.176 - 7.497]	0.819 [0.594 - 1.129]	0.518 [0.384 - 0.699]
NPH Insulin	3.212 [2.135 - 4.833]	1.041 [0.751 - 1.444]	0.506 [0.371 - 0.689]
Long-acting Insulin	14.333 [4.877 - 42.125]	0.140 [0.018 - 1.066]	0.279 [0.089 - 0.873]
Rapid-acting Insulin	3.980 [2.524 - 6.278]	0.304 [0.175 - 0.528]	0.935 [0.628 - 1.390]
Metformin only	1.216 [0.596 - 2.479]	0.964 [0.541 - 1.720]	0.929 [0.544 - 1.588]
Pregnancy Disorders			
Preeclampsia / Eclampsia	1.683 [0.544 - 5.207]	0.263 [0.060 - 1.153]	1.755 [0.652 - 4.724]
Maternal Delivery Complications			
Maternal Delivery Complications	1.538 [1.008 - 2.346]	1.299 [0.951 - 1.776]	0.645 [0.482 - 0.863]
Pre-term Delivery	0.705 [0.313 - 1.589]	0.482 [0.252 - 0.921]	2.126 [1.217 - 3.715]
Delivery < 28 weeks	0.000 [0.000 - Inf]	0.000 [0.000 - Inf]	30876333.671 [0.000 - Inf]
Assisted Delivery	1.579 [1.040 - 2.396]	1.360 [0.998 - 1.855]	0.611 [0.458 - 0.816]
Caesarean Section	1.622 [1.073 - 2.451]	1.389 [1.021 - 1.890]	0.591 [0.443 - 0.788]
Primary Caesarean Section	1.751 [1.167 - 2.627]	1.461 [1.076 - 1.983]	0.541 [0.406 - 0.721]
Secondary Caesarean Section	0.711 [0.274 - 1.841]	0.869 [0.448 - 1.687]	1.320 [0.715 - 2.440]
Vacuum Extraction	0.675 [0.154 - 2.962]	0.765 [0.273 - 2.149]	1.499 [0.584 - 3.851]
High Base Excess [a]	1.227 [0.457 - 3.291]	1.157 [0.529 - 2.532]	0.788 [0.374 - 1.658]
Low Base Excess [b]	0.554 [0.361 - 0.849]	0.782 [0.568 - 1.075]	1.664 [1.234 - 2.244]
In range Base Excess	1.737 [1.139 - 2.648]	1.251 [0.909 - 1.723]	0.622 [0.461 - 0.839]
Fetal Complications			
Fetal Complications	1.044 [0.693 - 1.572]	0.862 [0.629 - 1.182]	1.112 [0.830 - 1.491]
Birth Weight > 4000 g [c]	1.221 [0.597 - 2.499]	1.214 [0.691 - 2.131]	0.753 [0.437 - 1.296]
Birth Weight > 4500 g [c]	0.000 [0.000 - Inf]	1.368 [0.227 - 8.244]	1.380 [0.229 - 8.313]
LGA	1.553 [0.934 - 2.581]	1.486 [0.986 - 2.239]	0.545 [0.364 - 0.815]
SGA	0.750 [0.349 - 1.615]	0.828 [0.476 - 1.440]	1.346 [0.810 - 2.237]
Fetal Acidosis [d]	1.059 [0.619 - 1.810]	0.687 [0.443 - 1.066]	1.328 [0.897 - 1.965]
Admission to NICU	0.882 [0.424 - 1.834]	0.671 [0.380 - 1.188]	1.483 [0.891 - 2.469]
Anomalous APGAR 0 min [e]	1.172 [0.439 - 3.128]	0.524 [0.211 - 1.300]	1.524 [0.715 - 3.247]
Anomalous APGAR 5 min [e]	0.644 [0.081 - 5.130]	0.918 [0.235 - 3.580]	1.306 [0.366 - 4.667]
Anomalous APGAR 10 min [e]	0.000 [0.000 - Inf]	0.000 [0.000 - Inf]	34583644.643 [0 - Inf]
Anomalous APGAR [f]	1.172 [0.439 - 3.128]	0.524 [0.211 - 1.300]	1.524 [0.715 - 3.247]

[a]Base Excess > 2,
[b]Base Excess < -2,
[c]for gestational age at birth > 37 weeks,

[d]Arterial blood pH < 7.2,
[e]Anomalous if APGAR < 7,
[f]Anomalous APGAR at 0 or 5 or 10 min.
APGAR, appearance, pulse, grimace, activity, respiration; LGA Large for gestational age; NICU, neonatal intensive care unit; NPH, neutral protamine Hagedorn; SGA, small for gestational age

ESM Table 11: Odds Ratios (OR) and 95% confidence intervals for binary outcomes among the three clusters in the first partial model of the twofold cross-validation

	Cluster 1	Cluster 2	Cluster 3
Pharmacological Treatment (glucose-lowering medications)			
Drugs Prescribed - Insulin	4.534 [2.882 - 7.131]	0.397 [0.235 - 0.668]	0.590 [0.379 - 0.917]
Pregnancy Disorders			
Preeclampsia / Eclampsia	2.572 [1.162 - 5.693]	0.685 [0.284 - 1.649]	0.609 [0.269 - 1.379]
Maternal Delivery Complications			
Maternal Delivery Complications	0.998 [0.673 - 1.480]	0.847 [0.599 - 1.197]	1.163 [0.837 - 1.616]
Pre-term Delivery	1.143 [0.503 - 2.599]	0.995 [0.472 - 2.096]	0.913 [0.448 - 1.858]
Delivery < 28 weeks	0.000 [0.000 - Inf]	0.000 [0.000 - Inf]	67046105.266 [0.000 - Inf]
Assisted Delivery	1.006 [0.678 - 1.492]	0.779 [0.550 - 1.102]	1.247 [0.897 - 1.732]
Caesarean Section	1.125 [0.755 - 1.675]	0.900 [0.632 - 1.280]	1.013 [0.725 - 1.414]
Primary Caesarean Section	1.347 [0.844 - 2.151]	1.026 [0.669 - 1.573]	0.785 [0.520 - 1.185]
Secondary Caesarean Section	0.871 [0.529 - 1.434]	0.832 [0.538 - 1.286]	1.291 [0.862 - 1.934]
Vacuum Extraction/Forceps Delivery	0.651 [0.283 - 1.498]	0.563 [0.272 - 1.164]	2.092 [1.114 - 3.931]
High Base Excess [a]	3.297 [0.205 - 53.139]	0.000 [0.000 - Inf]	1.198 [0.075 - 19.268]
Low Base Excess [b]	0.660 [0.406 - 1.073]	1.167 [0.725 - 1.878]	1.206 [0.779 - 1.868]
In range Base Excess	1.473 [0.902 - 2.406]	0.888 [0.551 - 1.431]	0.823 [0.530 - 1.279]
Fetal Complications			
Fetal Complications	1.311 [0.859 - 2.001]	0.920 [0.641 - 1.321]	0.898 [0.636 - 1.269]
Birth Weight > 4000 g [c]	1.183 [0.682 - 2.052]	1.105 [0.675 - 1.808]	0.808 [0.501 - 1.303]
Birth Weight > 4500 g [c]	0.891 [0.187 - 4.252]	3.051 [0.85 - 10.951]	0.304 [0.064 - 1.445]
LGA	1.169 [0.746 - 1.830]	1.088 [0.729 - 1.623]	0.829 [0.564 - 1.218]
SGA	0.410 [0.142 - 1.180]	1.234 [0.620 - 2.457]	1.335 [0.685 - 2.600]
Fetal Acidosis [d]	1.306 [0.835 - 2.045]	1.037 [0.692 - 1.553]	0.798 [0.541 - 1.177]
Admission to NICU	1.831 [0.888 - 3.776]	0.482 [0.215 - 1.078]	1.135 [0.579 - 2.222]
Anomalous APGAR 0 min [e]	0.955 [0.404 - 2.254]	0.994 [0.472 - 2.096]	1.037 [0.512 - 2.101]
Anomalous APGAR 5 min [e]	1.190 [0.317 - 4.462]	0.658 [0.176 - 2.458]	1.248 [0.398 - 3.917]
Anomalous APGAR 10 min [e]	2.707 [0.598 - 12.258]	0.794 [0.153 - 4.129]	0.493 [0.095 - 2.562]
Anomalous APGAR [f]	0.850 [0.363 - 1.989]	1.134 [0.561 - 2.290]	0.993 [0.504 - 1.959]

[a]Base Excess > 2,
[b]Base Excess < -2,
[c]for gestational age at birth > 37 weeks,
[d]Arterial blood pH < 7.2,
[e]Anomalous if APGAR < 7,
[f]Anomalous APGAR at 0 or 5 or 10 min.
APGAR, appearance, pulse, grimace, activity, respiration; LGA Large for gestational age; NICU, neonatal intensive care unit; SGA, small for gestational age

ESM Table 12: Odds Ratios (OR) and 95% confidence intervals for binary outcomes among the three clusters in the second partial model of the twofold cross-validation

	Cluster 1	Cluster 2	Cluster 3
Pharmacological Treatment (glucose-lowering medications)			
Drugs Prescribed - Insulin	3.184 [1.971 - 5.144]	0.817 [0.522 - 1.279]	0.510 [0.324 - 0.803]
Pregnancy Disorders			
Preeclampsia / Eclampsia	1.396 [0.642 - 3.036]	1.421 [0.74 - 2.732]	0.550 [0.273 - 1.109]
Maternal Delivery Complications			
Maternal Delivery Complications	1.409 [0.912 - 2.175]	0.894 [0.638 - 1.253]	0.909 [0.653 - 1.265]
Pre-term Delivery	0.783 [0.265 - 2.313]	0.671 [0.289 - 1.561]	1.639 [0.753 - 3.566]
Delivery < 28 weeks	0.000 [0.000 - Inf]	0.000 [0.000 - Inf]	68110328.383 [0.000 - Inf]
Assisted Delivery	1.473 [0.955 - 2.270]	0.983 [0.702 - 1.377]	0.808 [0.581 - 1.124]
Caesarean Section	1.799 [1.171 - 2.762]	0.834 [0.594 - 1.170]	0.835 [0.599 - 1.163]
Primary Caesarean Section	1.288 [0.793 - 2.091]	1.200 [0.808 - 1.781]	0.712 [0.479 - 1.060]
Secondary Caesarean Section	1.708 [1.060 - 2.752]	0.621 [0.405 - 0.953]	1.091 [0.732 - 1.624]
Vacuum Extraction/Forceps Delivery	0.410 [0.144 - 1.168]	1.704 [0.931 - 3.118]	0.89’ [0.483 - 1.641]
High Base Excess [a]	0.791 [0.091 - 6.856]	3.467 [0.628 - 19.129]	0.263 [0.030 - 2.266]
Low Base Excess [b]	0.764 [0.454 - 1.288]	0.864 [0.553 - 1.349]	1.390 [0.889 - 2.175]
In range Base Excess	1.342 [0.790 - 2.279]	1.057 [0.669 - 1.671]	0.772 [0.490 - 1.216]
Fetal Complications			
Fetal Complications	1.453 [0.923 - 2.288]	0.823 [0.579 - 1.169]	0.966 [0.685 - 1.361]
Birth Weight > 4000 g [c]	2.584 [1.496 - 4.463]	0.518 [0.298 - 0.901]	0.902 [0.547 - 1.488]
Birth Weight > 4500 g [c]	2.330 [0.853 - 6.365]	0.604 [0.212 - 1.719]	0.829 [0.317 - 2.172]
LGA	2.113 [1.335 - 3.344]	0.731 [0.486 - 1.098]	0.814 [0.550 - 1.205]
SGA	1.026 [0.481 - 2.185]	0.770 [0.413 - 1.435]	1.253 [0.698 - 2.252]
Fetal Acidosis [d]	0.971 [0.591 - 1.595]	1.047 [0.709 - 1.546]	0.974 [0.664 - 1.429]
Admission to NICU	0.681 [0.259 - 1.793]	0.634 [0.308 - 1.304]	1.831 [0.943 - 3.555]
Anomalous APGAR 0 min [e]	2.127 [0.939 - 4.816]	0.987 [0.457 - 2.131]	0.566 [0.253 - 1.265]
Anomalous APGAR 5 min [e]	2.284 [0.413 - 12.64]	0.000 [0.000 - Inf]	2.599 [0.472 - 14.302]
Anomalous APGAR 10 min [e]	3.052 [0.504 - 18.499]	0.000 [0.000 - Inf]	1.942 [0.322 - 11.706]
Anomalous APGAR [f]	2.388 [1.083 - 5.267]	0.932 [0.435 - 1.999]	0.537 [0.242 - 1.194]

[a]Base Excess > 2,
[b]Base Excess < -2,
[c]for gestational age at birth > 37 weeks,
[d]Arterial blood pH < 7.2,
[e]Anomalous if APGAR < 7,
[f]Anomalous APGAR at 0 or 5 or 10 min.
APGAR, appearance, pulse, grimace, activity, respiration; LGA Large for gestational age; NICU, neonatal intensive care unit; SGA, small for gestational age

ESM Figures

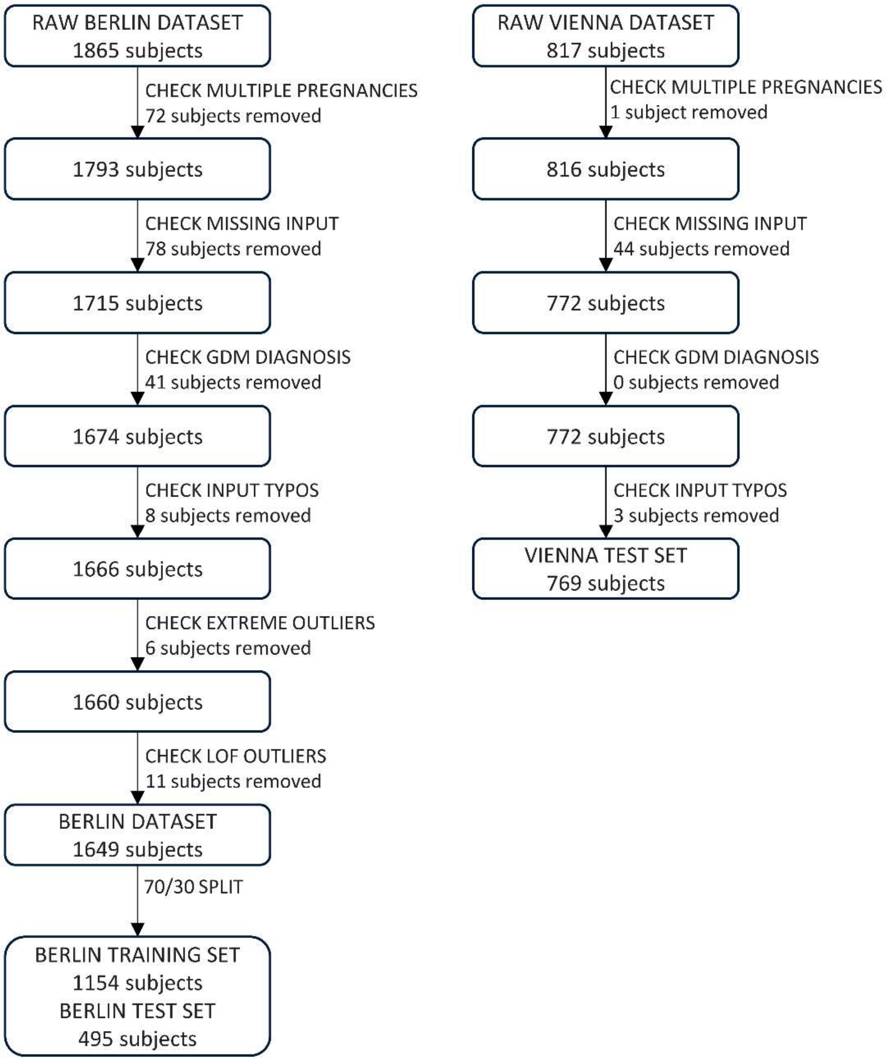

ESM Fig. 1: Diagram of the data pre-processing steps for the Berlin and Vienna datasets

The chart displays the sequence of operations carried out on the initial raw data, indicating the number of patients excluded at each stage. The remaining Berlin dataset was randomly split into training and test sets according to a 70/30 ratio, while Vienna dataset was utilised as an additional test set. GDM: gestational diabetes mellitus; LOF: Local Outlier Factor.

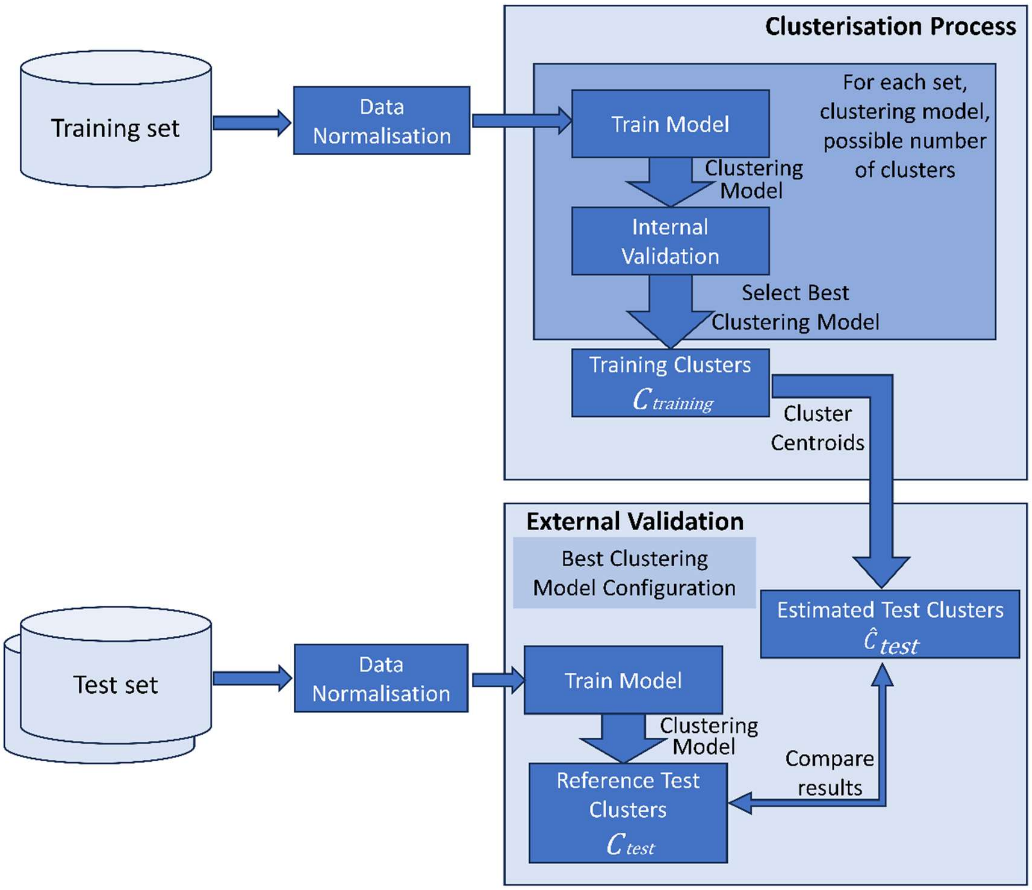

ESM Fig. 2: Diagram of the workflow for the development and validation of the GDM clustering model.
